# Combining *Shigella* Tn-seq data with gold-standard *E. coli* gene deletion data suggests rare transitions between essential and non-essential gene functionality

**DOI:** 10.1186/s12866-016-0818-0

**Published:** 2016-09-06

**Authors:** Nikki E. Freed, Dirk Bumann, Olin K. Silander

**Affiliations:** 1 Institute of Natural and Mathematical Sciences, Massey University, Auckland, New Zealand; 2 Infection Biology, Biozentrum, University of Basel, Basel, Switzerland; 3 Computational and Systems Biology, Biozentrum, University of Basel, Basel, Switzerland

**Keywords:** Gene essentiality, Tn-seq, Evolution, *Shigella*, *E. coli*

## Abstract

**Background:**

Gene essentiality - whether or not a gene is necessary for cell growth - is a fundamental component of gene function. It is not well established how quickly gene essentiality can change, as few studies have compared empirical measures of essentiality between closely related organisms.

**Results:**

Here we present the results of a Tn-seq experiment designed to detect essential protein coding genes in the bacterial pathogen *Shigella flexneri* 2a 2457T on a genome-wide scale. Superficial analysis of this data suggested that 481 protein-coding genes in this *Shigella* strain are critical for robust cellular growth on rich media. Comparison of this set of genes with a gold-standard data set of essential genes in the closely related *Escherichia coli* K12 BW25113 revealed that an excessive number of genes appeared essential in *Shigella* but non-essential in *E. coli.* Importantly, and in converse to this comparison, we found no genes that were essential in *E. coli* and non-essential in *Shigella*, implying that many genes were artefactually inferred as essential in *Shigella.* Controlling for such artefacts resulted in a much smaller set of discrepant genes. Among these, we identified three sets of functionally related genes, two of which have previously been implicated as critical for *Shigella* growth, but which are dispensable for *E. coli* growth.

**Conclusions:**

The data presented here highlight the small number of protein coding genes for which we have strong evidence that their essentiality status differs between the closely related bacterial taxa *E. coli* and *Shigella.* A set of genes involved in acetate utilization provides a canonical example. These results leave open the possibility of developing strain-specific antibiotic treatments targeting such differentially essential genes, but suggest that such opportunities may be rare in closely related bacteria.

**Electronic supplementary material:**

The online version of this article (doi:10.1186/s12866-016-0818-0) contains supplementary material, which is available to authorized users.

## Background

One general functional characteristic of a gene is essentiality - whether that gene is required for cellular viability and growth. In haploid (e.g. bacterial) genomes, this characteristic can be assessed by attempting to delete a specific gene from a genome. When such a deletion is not possible, this gene is frequently termed “essential” [1], implying that the gene is necessary for cell growth and viability. Gene disruption, although less precise, is more commonly used to infer essentiality using a similar criterion. For example, genes that cannot be disrupted by transposon insertion have been inferred as being essential (e.g. [2]).

One important question is how quickly essential functions change over evolutionary time. If orthologous protein coding genes in two bacterial strains differ in their essentiality classification, this suggests that either the biochemical nature of the protein has changed, or that the cellular context in which the protein acts has changed [3]. It has been experimentally established that such transitions can occur [4–6]. Here we examine how frequently proteins go from being essential to non-essential and vice versa in nature.

A recent study used Tn-seq to quantify changes in the essentiality classifications of protein coding genes between three alpha-proteobacteria: *Caulobacter crescentus*, *Brevundimonas subvibrioides*, and *Agrobacterium tumefaciens* [3]. The analysis showed that although orthologous cell components are well conserved, the essentiality of such components (e.g. those involved in the cell cycle) had changed considerably, with only 106 orthologous genes being essential in all three organisms, despite their relatively close evolutionary relationship (89–93 % identity in 16S RNA genes). This surprising result contrasts with those of Canals et al. [7], who performed a comparative Tn-seq analysis in the more closely related bacteria *Salmonella* typhimurium and *Salmonella* Typhi, and found 37 genes in which transposon density differed substantially between the two strains.

In this study we combine dense transposon mutagenesis with high-throughput sequencing (Tn-seq [8]) to quantify gene essentiality in *Shigella flexneri* 2a 2457T (hereinafter referred to as *Shigella*). We compare the essentiality classifications of protein coding genes in *Shigella* with a gold-standard assessment of essentiality in the closely related strain *Escherichia coli* K12 BW25113 (herineafter referred to as *E. coli*) [1]. These two stains are 99.5 % identical in their 16S RNA genes and share approximately 70 % of their genomic content.

This proximity in evolutionary distance, and the use of a gold-standard data set, brings two unique advantages that have not been available in other studies that have used Tn-Seq or similar methods to quantify gene essentiality [3, 7–20]. First, by relying on the null hypothesis that protein coding genes do maintain their essentiality characteristics, we can objectively assess which quantitative features in the *Shigella* Tn-seq data best predict essentiality or non-essentiality of their orthologous counterparts in *E. coli*; such a comparison to a gold-standard has not yet been used to assess the quality and sensitivity of Tn-Seq data [21], although several studies have validated a small number of Tn-seq-inferred growth defects using clean deletion methods (e.g. [11]). Second, the fact that these two taxa are very closely related taxa allows us to quantify on a short time scale the fraction of the essential gene complement that has changed, and thus the rate with which orthologues change in their essential functions.

The data presented here suggest that the essential gene complement of *Shigella* and *E. coli* overlap considerably. Indeed, we find no strong evidence that there are any protein-coding genes that are essential in *E. coli* but not *Shigella*. Conversely, we do find a small number of genes that play critical roles for *Shigella* growth, but which have dispensable roles in *E. coli*, or which are absent entirely from *E. coli.* This implies that the functional correspondence, in terms of essentiality, has changed for only a small number of protein-coding genes.

However, our analysis also suggests that some protein-coding genes that we observe as undisrupted by transposon insertions are in fact not essential for cell growth. Instead, they are either essential for transposon insertion to occur successfully, or their disruption (but not clean deletion) is detrimental to cell growth. This result emphasizes that in high throughput transposon mutagenesis studies, false positive inferences of essentiality may be common, and that simply increasing the resolution or precision of a dataset cannot necessarily solve this problem.

Taken together, our data suggests that the essential gene complement is relatively static over short time scales. However, when protein-coding genes do change from being non-essential to being essential, this appears more likely to occur in pathogenic organisms. One reason might be that the nutrient-rich host environment absolves the organism from manufacturing its own nutrients, leading to the loss of many catabolic functions [22]. If some of these functions are redundant, their loss will cause non-essential genes to become essential. It is also possible that such organisms have smaller population sizes and are prone to the accumulation of deleterious mutations. It would be interesting to see if this pattern is observed when comparing other pathogens to their free-living sister taxa. If antibiotics can be directed against the function or expression of such differentially essential genes, this may allow targeting such treatments toward specific bacterial strains.

## Results and discussion

### A transposon mutagenesis library provides fine-scale resolution of gene essentiality

**Fig. 1:**
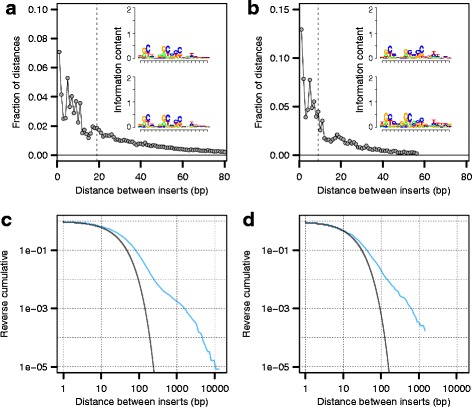
Histograms of distances between inserts for (**a**) *Shigella* chromosome and (**b**) *Shigella* virulence plasmid. The median distance between inserts is indicated by the dotted line. A plurality of inserts are separated by only a single bp for both in both the chromosome and the plasmid. The insets in (**a**) and (**b**) indicate the slight but detectable biases in transposon insert location using a weight matrix motif. The reverse cumulative plots show the observed fractions of distances between inserts for the chromosome (**c**) and the plasmid (**d**). In blue, the observed frequencies are plotted. In black, the expected frequencies are plotted, given a geometric distribution (negative binomial with the number of successes set to one) of inter-insert distances (see main text). For both the chromosome and the plasmid, there are considerably more large regions uninterrupted by transposons than one would expect given the geometric null model, observed as a shift of the curve to the right

We generated a transposon insertion library by conjugating a *Shigella icsA* mutant [23] with a strain containing a plasmid with an inducible mini-Tn10 transposase [24, 25] that has decreased hotspot activity ([26]; Fig. 1a, inset). After overnight growth of the library on Tryptic Soy Broth (TSB) agar plates, we harvested and pooled the colonies. On three separate days, aliquots of this pool were grown overnight in 2 mL of TSB, then diluted 1:100 and grown to an OD of 0.7 (see Methods). The resulting cells were harvested, the replicates were bar coded and sequencing libraries were prepared. We sequenced all replicates on a single Illumina HiSeq lane. For all the analyses presented in this study, we have pooled all data from these three replicates.

From this pooled data, we mapped insertions at 120,513 unique positions on the *Shigella* chromosome (with many insertions occurring on both the forward and reverse strands but at the same position), and 11,476 unique positions on the large *Shigella* virulence plasmid (see Methods). The median distance between inserts on the chromosome was 19 base pairs (bp); on the plasmid this distance was 9 bp. A plurality of inserts were separated by only a single bp (Figs. 1a and b), and 95 % of all inter-insert distances on the chromosome were less than 117 bp; the corresponding figure for the plasmid was 61 bp. Mapping these insertions also allowed us to infer SNP positions on the *Shigella* chromosome that have accrued during laboratory passaging, and SNP positions on the plasmid that differentiate the *Shigella flexneri* 2a 2457T plasmid and the *Shigella flexneri* 2a str. 301 plasmid pCP301 (see Methods).

**Fig. 2:**
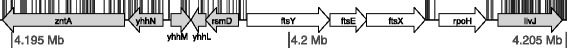
Orthologous genes known to be essential in *E. coli* are depleted for transposon insertions in *Shigella.* A region of the *Shigella* chromosome is shown, with genes whose orthologues are known to be essential for growth in *E. coli* (coloured in white) [1, 29], or non-essential (coloured in grey). The unique locations of transposon insertions are plotted as vertical black segments. In the genome region shown here, none of the genes essential in *E. coli* have orthologues that are interrupted in *Shigella*

**Fig. 3:**
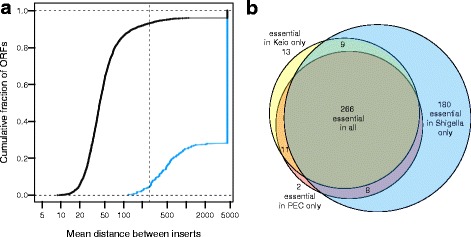
Differences in essentiality classification between *E. coli* K12 and *Shigella.* **a** Cumulative distributions showing the mean distances between inserts for ORFs depending on whether their orthologues are known to be non-essential (black curve) or essential (blue curve) in *E. coli*. All ORFs that are completely uninterrupted by transposons have been plotted at the very right end of the x-axis. The dotted vertical line indicates the cut-off that we used to delineate essentiality in *Shigella* (a mean distance between transposons of 260 bp or more). The 11 blue points to the left of the dotted vertical line indicate ORFs that are essential in *E. coli* but not *Shigella* by our metric. These are likely to be false negatives (i.e. essential in both *Shigella* and *E. coli*), as all have inter-insert distances greater than 100 bp (see main text). Black points to the right of the dotted vertical line indicate ORFS that we classify as essential in *Shigella* but not in *E. coli*. Many of these ORFs have *E. coli* orthologues whose deletion genotypes exhibit robust growth, suggesting that their essentiality status has changed. **b** A Venn diagram showing the overlap between essential orthologous ORFs in *E. coli* and *Shigella*

Although the distribution of transposon inserts was relatively even across both the chromosome (Additional file 1: Figure S1), at smaller scales we found many regions in which few or no insertions occurred (Additional file 2: Figure S2). Quantitative analyses showed that regions containing no transposon insertions for 100 bp or more were considerably enriched in the chromosome and to some extent, the plasmid, compared to a null model with geometrically distributed distances (Figs. 1c and d; see Methods). It is likely that many of these regions are critical for cellular growth in *Shigella*, or for maintenance of the plasmid. Indeed, we found that for many of the protein-coding genes in these regions, the orthologous *E. coli* genes are known to be essential (Figs. 2 and 3).

In total, eight out of 263 plasmid ORFs had no transposon insertions. Five relate to plasmid maintenance: three are replication related proteins (CP0258, CP0259, and CP0260), and two (CP0217 and CP0218) are located within the plasmid stabilisation region. The absence of inserts in these regions is explained by the fact that if such insertions did occur, the plasmid would be lost; such insertions would thus never be sequenced. One ORF, *acp*, is only 234 bp in length, and has a lower likelihood of harbouring insertions due to its small size. The two remaining ORFs lacking transposon insertions were *mxiH* (249 bp) and *spaS* (1026 bp), and both relate to antigen presentation. Indeed, we found that many of the ORFs most depleted for transposon insertions relate to the *mxi* and *spa* operons (Additional file 3: Table S1), but currently have no explanation as to why this was so.

Taken in conjunction, our data is largely consistent with the fact that the *Shigella* plasmid contains no essential genes [27], or genes whose deletion is detrimental for growth. Thus, our transposon library likely provides a fine-scale assessment of which *Shigella* chromosomal ORFs provide critical cellular functions.

### Average distance between inserts clearly delineates essential and non-essential ORFs

We next quantified which transposon insertion patterns in the chromosome were good predictors of the essentiality of open reading frames. To do so, we first identified 3,027 orthologous open reading frames present in both *E. coli* and *Shigella* for which we also had data on essentiality from both the Keio [1, 28] and the Profiling of the Escherichia coli Chromosome (PEC) [29] studies (Additional file 3: Table S1). We considered this combined gene set as a gold-standard of essentiality for two reasons: it is not subject to artefacts that might exist in Tn-seq dataset, such as insertion biases or biases arising during sequencing library preparation (e.g. [30]); and combining both the Keio and PEC datasets should result in few false positive or false negative essentiality characterizations. This set consisted of 277 orthologues considered essential by both studies [7], 2,718 genes considered non-essential by both studies, and 32 genes for which the two studies disagree.

These disagreements include ten genes considered essential by the PEC study alone and 22 considered essential by the Keio study alone. Some of these discrepancies may be due to genetic differences in the strains used for the two studies (MG1655 versus BW25113, respectively) [31]. Some of the other differences are likely due to methodology (single gene deletion versus large chromosomal deletions). For example, at least one gene that could not be deleted in the Keio set is an anti-toxin gene (*yefM* [1]; note that we do not consider that gene essential here).

Other differences may be due to duplications or other unforeseen genetic changes that occurred during the genetic manipulations, as has been noted previously [28]. Three pieces of evidence give some insight into how to resolve these discrepancies, and all suggest that the Keio data may be more prone to having both more false positives and false negatives. Considering gene conservation in gamma-proteobacteria: 90 % (9 out of 10) of the genes considered essential by the PEC study but not the Keio study have orthologues in more than two thirds of all surveyed gamma-proteobacteria [32]; only 40 % (13 out of 22) of the genes considered essential by Keio but not the PEC are similarly conserved (Additional file 3: Table S1). Similarly, 80 % of the PEC-specific genes have zero or one transposon insertion, while only 40 % of the Keio-specific genes are as depleted for insertions. Finally, for all variables we use here to predict essentiality (see below), the PEC data score better than the Keio data (Additional file 4: Figure S3.)

We next quantified several characteristics for each protein-coding gene in our Tn-seq data set, including the total number of inserts per ORF, the mean distance between inserts, the density of inserts, the length of the 5’ fraction of the ORF upstream of the first insertion, the largest uninterrupted region in the ORF, and others (Additional file 4: Figure S3). We took as a null hypothesis that generally, genes have maintained their essentiality characteristics since the divergence of *E. coli* and *Shigella*. We then tested which of these characteristics best predicted the essentiality status of their orthologous counterparts in the gold-standard dataset of open reading frame essentiality in *E. coli* (the Keio and PEC datasets).

We found that the best predictor of essentiality status in *E. coli* was the mean distance between transposon insertions in their *Shigella* orthologues (i.e. *L / (N*_*T*_*+1)*, with L being the ORF length and *N*_*T*_ being the total number of transposon insertions; Materials and Methods; Additional file 4: Figure S3). This metric is the inverse of the insertion index used by Langridge et al. (*N*_*T*_*/ L*), and as such, retains information on gene length when there are no insertions. For the *Shigella* orthologues of the 277 *E. coli* essential genes, only two had a mean distance between inserts of less than 150 bp. 11 (4 %) had a mean inter-insert distance less than 260 bp. In contrast, only 6.6 % of the orthologues of non-essential *E. coli* genes had a mean distance between inserts of greater than 260 bp (Fig. 3a). We selected this mean inter-insert distance of 260 bp as a cut-off for classifying *Shigella* ORFs as essential, as it provided a reasonable balance between protein coding genes classified as essential in *E. coli* but non-essential in *Shigella* (a 4 % false negative rate) versus non-essential in *E. coli* and essential in *Shigella* (a 6.6 % false positive rate). By extension, genes that are less than 260 bp in length and in which we do not observe insertions were inferred as essential (30 ORFs in total, of which 14 were ribosomal proteins and five were leader peptides). We note, importantly, that almost all of the predictors we tested performed extremely well (Additional file 4: Figure S3.).

We also mapped insertions in 25 out of 99 tRNAs. 22 of these harboured only one or two insertions, and all had three or fewer except the tRNA for selenocysteine, which contained seven transposon insertions (Additional file 5: Table S4). However, as neither the Keio nor the PEC dataset analyse the essentiality of tRNA genes, we did not analyse this data further.

We next investigated in greater detail the disagreements in essentiality classification between *E. coli* and *Shigella* (Fig. 3b). Of the 11 *E. coli-*essential genes that this metric identified as non-essential in *Shigella*, all are likely to be false negatives (i.e. in fact essential in *Shigella,* but not classified as such by our criterion). Nine have a mean distance between inserts of greater than 150 bp (Fig. 3a). Four contain only a single insertion, and all eleven contain six or fewer insertions. This suggests, surprisingly, that there are no genes that are essential in *E. coli* but whose *Shigella* orthologues are non-essential*.* This similarity in essentiality is not due to the fact that we use a characteristic that most closely predicts essentiality in the gold standard dataset – this result is robustly corroborated by any meaningful metric that we used (e.g. using other mean distances between inserts as cut-offs for essentiality, using the total number of inserts, the longest uninterrupted gene fraction, or others (Additional file 4: Figure S3)). Overall, this data gives us a strong reason to believe that genes will have maintained their essentiality status (or near-lethal effects on growth) since the divergence of *E. coli* and *Shigella*.

### Many non-essential *E. coli* orthologues of essential *Shigella* genes exhibit impaired growth

**Fig. 4:**
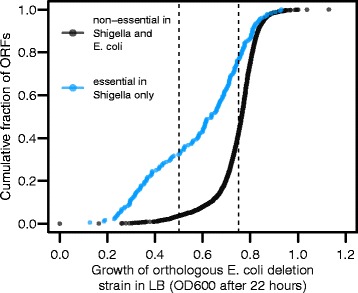
Growth yields of orthologous gene pairs. Orthologous gene pairs that are non-essential in *E. coli* but inferred as essential in *Shigella* (blue) tend to exhibit low growth yields in *E. coli*. ORFs that we infer to be uniquely essential in *Shigella* consistently have *E. coli* orthologues with low growth phenotypes in LB media after 22 h (apparent as a strong leftward shift in the cumulative curve). For genes inferred as uniquely essential in *Shigella,* 32 % of the orthologous *E. coli* deletion genotypes exhibit low growth yields (less than 0.5 OD600 after 22 h of growth in LB). For genes we classified as non-essential in *Shigella* and *E. coli* only 3.6 % exhibit low growth yields. Thus, some genes we infer as essential in *Shigella* may not be strictly essential, but instead be required for robust growth. Despite this enrichment for low-growth phenotypes, there are many genes which we infer as essential in *Shigella*, but which have *E. coli* orthologues whose deletion genotypes exhibit robust growth (OD600 greater than 0.75 after 22 h growth in LB)

The above evidence for the rarity of changes in gene essentiality classification suggests that many of the discrepancies in essentiality between *E. coli* and *Shigella* are false positives due simply to the *Shigella* mutants being non-essential, but having significantly impaired growth. Indeed, of the 180 discrepant genes classified as non-essential in *E. coli* but essential in *Shigella*, 32 % of the orthologous *E. coli* deletion genotypes exhibit low growth yields (less than 0.5 OD600 after 22 h of growth in LB [1]). This contrasts strongly with the 2557 ORFs we classified as non-essential in *Shigella*: only 3.6 % of the orthologous *E. coli* deletion genotypes had low growth yields (Fig. 4). Similar but less striking patterns were observed for growth in glucose minimal MOPS media (Additional file 6: Figure S4).

It is important to note that Tn-seq assays have only limited power to differentiate essential genes from those whose deletion results in severe growth deficiencies. During the course of preparing the library for sequencing, we estimate that at least 15 generations of growth occurred. If a mutant has a growth rate even 40 % that of the wild type, we would expect it to undergo only six doublings in contrast to the 15 of the wild type. This would result in a greater than 500-fold underrepresentation of such a mutant (2^6^/2^15^). In addition, this calculation does not take into account any effects that the mutations have on the length of the lag time, which might also have significant effects on the relative frequency of some mutants. If, in contrast, transposon insertion affect only the growth yield, then this will have much less of an effect.

In light of this limited resolution power; given that our prior expectation is that essentiality status changes only rarely; and because we are specifically interested in genes that may have changed in essentiality status, from this point on we focus our analysis on essential *Shigella* genes whose orthologous *E. coli* deletion genotype exhibits robust growth yields (OD600 greater than 0.75 after 22 h growth in LB (Fig. 4)). For these genes, we have relatively high confidence that while their deletion in *E. coli* has few effects on growth, their disruption in *Shigella* is lethal or results in a severe growth deficiency.

### Artefacts of the transposon screen explain some false positive discrepancies

Forty-two essential *Shigella* genes have orthologous *E. coli* deletion genotypes with growth yields higher than 0.75 OD600 after 22 h growth in LB [1] (Fig. 4). Careful inspection suggested that some genes were present in this set due to differences in the growth conditions between *E. coli* Keio and PEC collections and our own. For example, *fhuACD*, and *tonB* all appeared in this set of genes (Table 1). All four are involved in iron acquisition, and it is likely that iron was limiting in the solid agar media [33] used during the preparation of the Tn-seq library, as compared to the liquid LB used to measure growth in the Keio study.

**Table 1:** Genes artefactually inferred as essential in *Shigella*

Gene(s)	Evidence for Artefactual Inference of *Shigella* Essentiality
*acrAB, tolC, ybaB, ksgA, yebC, smpB* (0.73) ^a^	Affect kanamycin resistance [35–37]
*dnaQ, holD, recD*, *xseA*, *ruvA* (0.58), *ruvB* (0.60), *ruvC* (0.61), *recB* (0.58), *recC* (0.65)	Likely to affect the transposition process; *dnaQ, holD, ruvA,* and *ruvB* inferred as essential using Tn-seq in *Salmonella* [14]
*priB*	Deficient in plasmid maintenance [63, 64]
*fhuACD, tonB*	Involved in iron acquisition which is critical for growth in the iron limited media used in this study [33]
*rpsT*	S20 ribosomal subunit; new data indicates mutants have poor growth [42] (in conflict with Keio data)
*miaA*	tRNA dimethyl transferase; previous data indicates *E. coli* mutants have poor growth [43] (in conflict with Keio data)
*ompA*	Outer membrane porin; clean knockouts appear viable [65]; mutant forms are frequently lethal [44]
*pitA*	Metal phosphate transporter with ten transmembrane segments; transposon disruption of substrate transporters is three-fold more likely to be inferred as essential compared to clean deletion (see main text; Fig. 5)
*potB*	Type I ABC transporter (Putrescine / spermidine transporter); see above
*sapB*	Type I ABC transporter (unknown substrate); see above
*ptsH, yeaC, ydhR*	Short reading frames (306 bp, 272 bp, and 258 bp, respectively)

^a^Gene deletions of orthologous *E. coli* genes with growth levels less than OD600 0.75 have these levels in parentheses

Listed here are those genes that were likely inferred as essential largely due to the gene disruptions having direct effects on (1) antibiotic resistance, (2) successful transposition events, (3) differences between the growth conditions of the *E. coli* and *Shigella* essentiality studies, or (4) systematically different effects of gene disruption versus gene deletion.

For a second set of genes, the discrepancies are likely due to the differences in methodology between the *E. coli* (precise gene deletions) and *Shigella* (transposon inactivation) studies (Table 1). We inferred *acrAB* and *tolC* as essential. These genes act together as an efflux pump, and mutations in these genes result in hypersensitivity to antibiotics [34]. Thus, clones with transposon insertions in these genes are unlikely to survive during library growth under kanamycin selection. A similar explanation likely underlies the fact that we inferred *ybaB, ksgA, yebC,* and *smpB* as essential: these four play role in aminoglycoside resistance [35–37].

We also inferred *priB*, *dnaQ*, *holD*, *xseA*, and *recD* as being essential in *Shigella*, although the *E. coli* deletion genotypes exhibit robust growth. All of these are involved in DNA replication, recombination, and double strand break repair, all of which are essential processes in the completion of the transposition process [38]. The related genes *recBC* and *ruvABC* contained a single insert between the five of them, while the *E. coli* deletion genotypes all exhibit only slightly impaired growth of 0.6 OD600 or more (Table 1). Certain *recBC* mutants can have considerable effects on the rate of Tn10 excision [39, 40] and we speculate that this may be one reason why we rarely observed insertions in these loci. It has also been speculated that *ruv* mutants inhibit transposition [41]. We propose that after transposition occurs, in order for the event to be successfully resolved, transcription of these genes is often required, and the transposition itself precludes the formation of a proper transcript.

Thus, the dispensability of these ten genes in *E. coli*, and the similarity in their function, suggests that they all affect successful transposon insertion rather than having critical effects on growth. Notably, *priB*, *dnaQ*, *holD, ruvA,* and *ruvB* were also inferred as essential in the closely related bacterium *Salmonella* typhimurium via a high-throughput transposon assay. In the same study *ruvC, recB, recC,* and *ybaB,* were inferred as extremely important for growth while *ksgA* and *yebC* were inferred as significantly impairing growth [14]. Again, the majority of these knockouts in *E. coli* exhibit very robust growth (greater than 0.75 OD600 after 22 h growth in LB)*.* Given the roles that these genes are known to play in transposition and antibiotic resistance, this suggests that the inference of essentiality may be due to artefacts of the transposon screen.

For a third set of genes, the literature presents conflicting information on the growth phenotypes, with studies that have individually assessed growth rates suggesting poor growth. These include *rpsT* [42], *miaA* [43], and *ompA* [44] (Table 1).

There were also three open reading frames that we inferred as differentially essential, as they were completely uninterrupted in our data. However, these open reading frames, *ydhR*, *yeaC*, and *ptsH*, are small and thus less likely to be disrupted, being 306 bp, 270 bp, and 258 bp long, respectively. Under our theoretical geometric distribution fit (see Methods), one in 1,400 gaps will be longer than 150 bp and one in 170,000 will be longer than 250 bp (Fig.1c). Insertion biases (Fig. 1a, inset) will increase these frequencies. Given that we observe over 100,000 insertion sites (and thus gaps), it is probable, but not certain, that this discrepancy is not driven by different physiological roles that they play in *E. coli* as compared to *Shigella*.

**Fig. 5:**
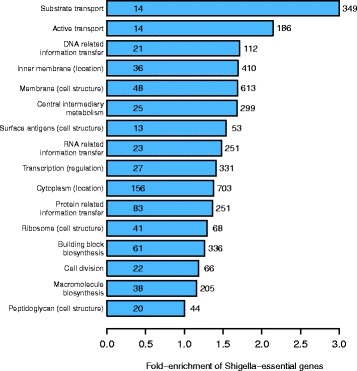
Functional categorization of transposon disrupted genes. Transposon disruption of *Shigella* genes with transport-related functions are more likely to be inferred as essential compared to clean deletions of similarly functioning genes in *E. coli*. We classified genes according to function using the MultiFun functional classification system [62]. For any category containing more than ten essential *E. coli* genes, we also calculated the number of *Shigella*-essential genes. As expected, most categories show a relative excess of *Shigella*-essential genes, as we inferred approximately 50 % more genes as being essential in *Shigella* versus *E. coli* (Fig. 3b). However, two functional categories show a clear excess above this level: substrate transport and active transport, showing a 3.2- and 2.2-fold increased probability of inferring a gene as being essential in *Shigella* as opposed to *E. coli*. This provides evidence that genes in these functional categories are more likely to be inferred as artefactually essential. This artefactual effect has not been noted previously. For each functional category (y-axis), we show the number of genes in that category (to the right of each bar); the number of genes found to be essential in *E. coli* (within each bar); and the level of enrichment of essential genes in *Shigella* (x-axis)

Finally, we tested for other possible artefactual patterns in the data based on gene function. We asked whether there were specific functional categories in which genes were more likely to be inferred as essential using the transposon mutagenesis screen in *Shigella* as opposed to clean deletions in *E. coli*. We found two functional categories of genes that showed clear enrichment: genes involved in substrate transport and / or active transport, which were 3.2- and 2.2-fold enriched, respectively (Fig. 5). We hypothesize that one reason for this enrichment is that truncated versions of these proteins disturb the operation of the *sec* machinery, thereby decreasing or stopping growth. Thus, we propose that the four active transporters we infer as essential in *Shigella* but not *E. coli* (Table 1) are artefacts due to the transposition process resulting in truncated proteins.

**Fig. 6:**
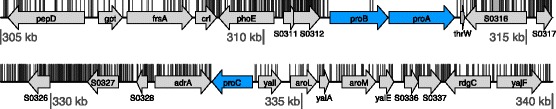
Three genes involved in proline biosynthesis (*proABC*) appear uniquely essential in *Shigella*. The orthologous *E. coli* deletion strains exhibit robust growth (OD600 greater than 0.75 after 22 h growth in LB), but are essential by our criteria. *proA* and *proC* completely lack transposon insertions, while *proB* contains only two insertions near the 3′end, which leaves approximately 70 % of the gene intact, including the entire kinase and substrate-binding domain

### Genes uniquely essential in *Shigella flexneri*

While many differences in essentiality classification between *Shigella* and *E. coli* are likely due to (1) severe growth defects present in both *E. coli* and *Shigella* rather than strict essentiality; and (2) differences in environmental conditions (e.g. iron) between the *E. coli* and *Shigella* assays; and (3) artefacts of the *Shigella* transposon screen that do not occur in the *E. coli* knockout screen, we do find a number of genes which we infer to be uniquely essential or important for growth solely in Shigella. We expect that the physiological differences between *E. coli* and *Shigella* drive these differences in gene essentiality (Table 2).

**Table 2:** Genes inferred as uniquely essential in *Shigella*

Gene(s)	Function [49]	Support for Different Physiological Roles in *E. coli* and *Shigella*
*lysS*	aminoacyl tRNA synthetase, tRNA modification	The *lysU* functional homologue is absent in Shigella [45]
*proABC*	proline biosynthesis	The active proline transporter *putP* is absent from Shigella [46]. The cryptic transporter *proY* may be silent, as observed in Salmonella [47], possibly necessitating proline biosynthesis
*ackA* *pta* *aceEF* *pykF*	acetate kinase phosphotransacetylase pyruvate dehydrogenase pyruvate kinase	All affect acetate accumulation [66] and utilization [23], which is required for robust growth (Shigella lacks the acetyl CoA synthetase present in *E. coli* K12 [67])
*rfbA, rfbF, rfbG, rfc,* and *rfbI*	sugar nucleotide biosynthesis for LPS	All except *rfbA* lack *E. coli* K12 orthologues, as this locus has been replaced by the laterally transferred *wbb* locus [48]
*cysM*	cysteine synthase B	ORFs in cysteine synthase A also depleted for inserts; 4 out of 5 sulfate ABC transporters depleted for inserts (see main text)
*tufB*	elongation factor EF-Tu	*tufB* and *lepA* (next entry) both are involved in translation elongation; the other ORF involved, *tufA,* is depleted for inserts (see main text)
*lepA*	elongation factor 4	See above
*spr*	murein DD-endopeptidase	None known
*rsxB*	soxR-reducing complex	None known

These genes were inferred as essential in *Shigella*, but have orthologous *E. coli* deletion genotypes that exhibit robust growth (greater than 0.75 OD600 after 22 h growth in LB). The genes in the *rfb* operon have no orthologues in *E. coli* K12 (see main text)

Among the set of genes essential in *Shigella* but dispensable in *E. coli* is *lysS*: this ORF has a functional homologue in *E. coli* (*lysU* [45]), while in *Shigella flexneri* there is no homologue. Also in this set of genes are *proA, proB, and proC* (Fig. 6). These genes act in proline biosynthesis. Given the rich media the cells were grown in, it is surprising that they would be essential. In addition, as *proB* is involved in the first committed step of proline synthesis, its disruption should not cause accumulation of toxic intermediates. However, the data provide strong evidence that the disruption of any these three genes is either lethal or causes severe growth defects (Additional file 7: Figure S5). Interestingly, the active proline transporter *putP* is absent from *Shigella* [46]. It is also known that in *Salmonella,* the cryptic proline transporter *proY* is silent [47], and we hypothesize that this may also be true of this transporter in *Shigella.* Thus, inefficient proline transport from the media might necessitate biosynthesis.

A suite of genes involved in acetate utilization (*aceE*, *aceF*, *ackA*, *pta*, and *pykF*) were all inferred as essential in *Shigella* but dispensable in *E. coli.* The significantly detrimental effect on growth that such mutants have has been noted previously using a completely different approach [23]. The difference in essentiality between these two organisms is most likely due to the absence of acetyl CoA synthetase from *Shigella,* and confirms the sensitivity and relevance of our transposon mutagenesis assay for assaying differences between *E. coli* and *Shigella* biology.

We found one gene involved in cysteine biosynthesis, *cysM* (coding for cysteine synthase B), which was essential in *Shigella* but not *E. coli.* Notably, *cysK* and *cysE*, which code for cysteine synthase A, have relatively large inter-insert distances (139 bp and 206 bp, respectively), despite having fairly robust growth in *E. coli*. This suggests that cysteine availability may limit growth in *Shigella.* Further supporting this hypothesis is that ORFs involved in cysteine import have fewer than expected inserts: of the five sulfate / thiosulfate ABC transporters, three (*cysA*, *cysW*, and *cysU*) have either zero or one insert; a fourth (*cysP*) has only five inserts. All of these have growth yields of OD600 0.67 or greater in *E. coli*. It is also possible, however, that the low number of inserts in these genes is due to the disproportionately deleterious effects that transposon insertions seem to have in membrane transport proteins, as suggested above.

For only four other orthologous gene pairs is there strong evidence of discrepant essentiality status: *tufB* (no insertions over 1183 bp; Additional file 7: Figure S5), *lepA* (five insertions over 1800 bp), *rsxB* (one insert at base pair 66 across 576 bp), and *spr* (one insert at base pair 543 across 567 bp). Both *tufB* and *lepA* are involved in translation elongation, and interestingly, we also found very few transposon insertions in the only other non-essential gene involved in elongation, *tufA* (two inserts; Additional file 7: Figure S5). This suggests that genes involved in translation elongation are important for *Shigella* growth despite their relative dispensability in *E. coli* (0.72 OD600 after 22 h in LB). We note that *tufA* and *tufB* are nearly identical in their sequence, which can create ambiguities in mapping some reads. However, this does not explain the absence of reads mapping to either of them. Understanding the molecular mechanisms driving these apparent disparities in growth phenotypes between *Shigella* and *E. coli* is an important topic for future research. At this point we have no convincing functional connections for the remaining two differentially essential ORFs, *rsxB* and *spr*.

Finally, the transposon insertion data indicated that within a single large operon, containing the ORFs *rfbACEFGI / rfc,* four genes completely lacked insertions (*rfbF, rfbG, rfc,* and *rfbI; rfbA* had a single insertion) (Additional file 8: Figure S6). Only *rfbA* and *rfbC* in this operon have *E. coli* orthologues. The remaining genes lie within a commonly laterally transferred region of the *E. coli* chromosome containing *wbbHIJKL, wzxB* (*rfbX*), and *glf*. These were all laterally transferred into the K12 lineage [48], replacing the *Shigella*-like *rfb* operon. The genes in this operon all play a role in sugar nucleotide biosynthesis necessary for O-antigen synthesis and production of the lipopolysaccharide component of the outer membrane [49]. The functionally related proteins with *E. coli* orthologues, *waaJ* (*rfaJ* in *E. coli*) and *waaD* (*wbbK* in *E. coli*), are also depleted for transposon insertions, having one and two inserts, respectively. This provides evidence that specific aspects of this process have become essential in *Shigella*, despite these genes having been replaced by a laterally transferred set in *E. coli* K12.

## Conclusions

By exploiting the extremely close evolutionary relationship of *Shigella flexneri* with *E. coli* K12, the bacterial strain that has been the most extensively and carefully characterized for its essential gene complement [1, 29], we were able to develop an objective metric to precisely quantify how the results of the Tn-seq data relate to essentiality.

A superficial analysis of our Tn-seq data suggested that a total of 481 ORFs in *Shigella* were essential for cellular growth in rich media. This is very much in line with what other Tn-Seq studies have found, with numbers ranging from 480 in *Caulobacter crescentus* [9] to 447 in *B. subvibrioides* to 372 in *Agrobacterium tumefaciens* [3]. However, it is considerably more than the number found in *E. coli* using precise in-frame gene knockouts [1] or in *Bacillus subtilis* using targeted knockdowns [50], which is on the order of 300 essential genes. In addition, we found that close to 100 % of the reading frames that were classified as essential in *E. coli* K12 were also essential in *S. flexneri*, giving us a strong prior expectation that the essentiality classifications should match between these two taxa.

A more nuanced analysis suggested four explanations for artefactual discrepancies in essentiality between *E. coli* and *Shigella*: (1) many *Shigella* genes were not strictly essential but instead gene disruption caused severe growth impairment; (2) differences in experimental conditions (e.g. iron availability); (3) many of the genes we inferred as essential were important for antibiotic resistance or successful transposition, and are in fact dispensable for growth; and (4) transposon disruption of specific functional classes of genes may result in systematically different effects as compared to gene deletions, for example due to the production of truncated protein products. By carefully dissecting the functions of discrepant genes that do not appear to be artefactual, we were able to pinpoint several genes for which there is evidence of differential physiological roles in *E. coli* and *Shigella*. Among others, these included *lysS,* three genes involved in proline biosynthesis, and a suite of genes involved in acetate utilization (Table 2). In addition to these, we found one large operon which appears to have an essential role in *Shigella* growth but which is missing completely in *E. coli.* There is also some evidence that cysteine biosynthesis or transport is limiting in *Shigella*, or under the conditions we imposed during the experiment, as several genes relating to these process have fewer transposons than would be expected.

Even after attempting to decrease false positive inferences of gene essentiality in *Shigella*, it appears to be considerably more common for genes to be dispensable for growth in *E. coli*, but critical for growth in *Shigella*. We suggests that one reason *Shigella* more may have a larger complement of essential genes than *E. coli* is that it frequently lives as an intracellular pathogen, and may have lost some of the functional redundancy that is present in *E. coli*. This may occur because host environments provide an abundance of nutrients, or because pathogens requiring a small infectious dose, such as *Shigella* [51], have inherently smaller population sizes and are more subject to genetic drift. A third possibility is that changes in gene function or redundancy may have occurred through selection for increased virulence, which has resulted in the inactivation of certain genes being selectively advantageous. Finally, we note that the discrepancies in essentiality between these two bacteria may be exploited to develop antibiotics that have strain-specific effects [23].

## Methods

### Strains

For all experiments*, Shigella flexneri* 2457T ∆*icsA*, provided by M. B. Goldberg, was used as the parental strain. This strain is unable to exploit the host actin cytoskeleton for motility and spreading [52]. Bacterial cells were grown in Tryptic Soy Broth (TSB) media.

### Transposon library construction

Using a Tn10 transposon with a T7 promoter [24, 25] we created a transposon insertion library by mating *E. coli* strain BW20767 containing the pJA1 transposon plasmid with a spontaneous nalidixic acid resistant clone of *Shigella flexneri* 2457T Δ*icsA*. 1 mL of overnight culture of each strain was placed on a 0.45 micron nitrocellulose filter and allowed to grow on TSB agar plates for 5 h at 37 °C (with no antibiotic selection). Each filter was then placed into 2 mls of TSB media in a 15 ml conical vial and vortexed to remove the bacteria from the filter. 20 mM isopropyl-β-D-thiogalactoside (IPTG) was added to induce transposase expression. Mated bacteria were plated onto 150 mm TSB agar plates containing the appropriate antibiotics, and colonies were allowed to grow at 37 °C for 18 h. Four matings were performed, resulting in a total of 38 agar plates. We estimate that each plate contained more than 10^4^ colonies. All colonies from these plates were pooled, and 100 μl aliquots of the transposon library were stored at -80 °C.

Using these pooled aliquots, three replicate experiments were carried out on different days: one aliquot of the transposon library was grown for 18 h at 37 °C in TSB, and then diluted 1:100 and grown to exponential phase (0.7 OD600). DNA was extracted from the pelleted cells using the Bacterial Genomic Miniprep Kit (Sigma).

### Sequencing

To amplify the transposon region from these pools, we used one top strand primer annealing to the transposon and a pool of three bottom strand primers each of which consisted of 10 random nucleotides followed by a pentamer of common nucleotides in *E. coli* [53]: N_10_GGTGC, N_10_GATAT, and N_10_AGTAC, using Phusion pfu (Additional file 9: Figure S7). A nested PCR was then performed to add the P7 and P5 Illumina adapters, as well as a barcode. The products from this second PCR were then size selected for inserts between 200 bp and 300 bp, quantified using a Qubit, and sequenced on an Illumina HiSeq2000 at the D-BSSE Quantitative Genomics Facility, resulting in 49 bp single end reads. We used a custom sequencing primer on the P5 end of the molecule such that on both ends of the molecule, reads started directly on the chromosome.

### Read mapping

In total, we obtained 115,677,316 reads. We found that the number of reads at each location in the genome varied by up to four orders of magnitude. For this reason, we considered only whether an insertion had occurred at a specific location, and not on the number of reads we obtained at a specific location, which is likely to be highly biased due to PCR artefacts. We thus first deduplicated the reads using tally [54], and then used bowtie2 [55] to align the reads to the *Shigella flexneri* 2a 2457T genome and the *Shigella flexneri* 2a str. 301 plasmid pCP301. The sequence of the *S. flexneri* 2457T plasmid is not available. However, the *S. flexneri* 2457T and 2a str. 301 plasmids are nearly identical in sequence (differing by 30 SNPs; see below). Sequence reads were not trimmed for quality as read quality is taken into account in bowtie2. We used the --sensitive-local option to allow soft clipping on the 3′ end of the reads (so that reads that contained adapter sequences at the 3′ end could map successfully), and required at least 22 bp of perfectly matching sequence at the 5′ end of the read.

### SNP inference

We checked for single nucleotide polymorphisms (SNPs) on both the chromosome and the plasmid using the samtools mpileup and bcftools utilities [56, 57]. We retained as possible SNPs only those sites that fulfilled the following three criteria: (1) the SNP was inferred as homozygous (necessarily true, as *Shigella* is haploid); (2) the quality score was above 20; and (3) at least three reads on both the reverse and forward strands confirmed the SNP. We found 99 SNPs on the chromosome (as compared to the reference *Shigella flexneri* 2457T in NCBI) and 30 SNPs on the plasmid (as compared to the *Shigella flexneri* 2a str. 301 plasmid in NCBI (in addition to 12 and 2 small indels, respectively). These are detailed in Additional file 10: Table S2 and Additional file 11: Table S3, respectively.

Within chromosomal protein coding regions, 44 % of all SNPs were synonymous, while 32 % fell outside of genic regions (i.e. protein coding or RNA genes). These fractions are greater than one would expect if such SNPs were randomly located on the genome. Only 24 % of all mutations in chromosomal coding regions are expected to be synonymous (not accounting for mutational biases), and only 28 % of the chromosome is annotated as nongenic (including repeat regions, although for many of these regions, the absence of an annotation may be erroneous). Additionally, 10 of the 12 (83 %) small chromosomal indels fell outside coding regions. This suggests that during the culturing and derivation of the *Shigella flexneri* 2a 2457T *virG* mutant there was some selection against nonsynonymous substitutions and coding indels. More importantly, the small number of SNPs that we found suggests that few reads remained unmapped due to sequence differences between the strain used in our experiments and the sequenced GenBank strain.

### Inferring transposon insertion locations

In total, the reads mapped to 80,712 unique locations on the forward strand and 75,568 on the reverse strand of the chromosome, for a total of 156,280 insertions. Some of these insertions occurred at identical positions but on opposite strands, so in total, insertions occurred at 120,513 unique sites in the chromosome. Correspondingly, the reads mapped to 7,469 unique locations on the forward strand and 7,827 unique locations on the reverse strand of the plasmid, and a total of 11,476 unique sites.

During the insertion of the Tn10 transposon, a 9 bp target DNA sequence is duplicated [58]. We accounted for this duplication in calculating the distances between insertions (by moving the inferred site of insertion for one direction (we arbitrarily selected the antisense direction) backward by 9 bp). Similarly, this duplication was accounted for in calculating various statistics of insertions within genes: sense insertions that were inferred as occurring in the last 9 bp of a gene were ignored in calculating the mean number of insertions per gene (as these bp are duplicated upstream of the insertion). Antisense insertions occurring in the first 9 bp of a gene were ignored, as these bp are duplicated downstream of the insertion.

Using the read frequencies at all unique insert locations, we found that the transposon insertions occurred in a slightly biased manner, integrating more often at sites similar to the known 9 bp consensus NGCTNAGC [58], although this bias was relatively weak (Figs. 1a and b, insets). This low level of bias is likely due to our using a transposon with reduced hotspot activity [26]. In addition, we found that insertion frequency was slightly influenced by nucleotides further downstream of this 9 bp consensus (Figs. 1a and b, insets). Sequence logos for this analysis were visualized using the R package seqLogo [59].

As expected given the variation in insertion densities across the chromosome, we found high variance in the distribution of inter-insert distances. The total length of the *S. flexneri* genome is 4,599,354 bp in total. Given that we observed 120,513 inserts, under a model of random insertion, we would expect a median distance between inserts of 26 bp, with 95 % of all inter-insert distances being less than 112 bp (under the assumption that these distances are distributed in a geometric manner (i.e. a negative binomial with the number of successes set to one). For the plasmid, we observed 11,476 inserts over 221,618 bp, such that we expect a median distance of 13 bp between inserts, and that 95 % of all inter-insert distances are less than 56 bp. However, as noted above, we found that on average transposons insertions were separated by a median of 19 bp on the chromosome and 9 bp on the plasmid. We fit a geometric distribution to the observed data over 95 % of the range of the inter-insert distances (i.e. from 1 to 117 bp for the chromosome and from 1 to 61 bp for the plasmid) to more exactly quantify this over-dispersion (Fig. 1c and 1d, respectively).

### Essential open reading frames

We identified 3,027 unambiguously ORFs that were present in both *E. coli* and *Shigella flexneri* 2457T [32], and for which we had essentiality data. We used reciprocal shortest distance [60] to find orthologues, with the requirement that the alignment of the two hypothetical orthologues extend over at least 60 % of the longer ORF. To establish a gold-standard set of essential genes we combined the data from two studies of the effects of gene deletion on growth in *E. coli* K12: the Keio collection [1] and the PEC study [29]. We retained only those ORFs which we had data on essentiality from both studies. We then quantified which transposon insertion patterns that most closely corresponded with the essentiality delineations in theses studies. Specifically, we selected the feature that maximized the number of true positive essential genes (maximizing the sensitivity) while minimizing the number of FP (maximizing specificity) (this metric is a receiver operator characteristic for which we quantified the area under the curve (AUC; Additional file 4: Figure S3)). We selected from eleven non-independent features: (1) the total number of insertions; (2) the mean number of bp between insertions; (3) the median number of bp between insertions; (4) the number of bp in the 5′ end preceding the first insertion; (5) the number of bp in the 5′ end preceding the first insertion relative to the total bp in the gene; (6) the number of bp in the 5′ end preceding the second insertion; (7) the number of bp in the 5′ end preceding the second insertion relative to the total bp in the gene; (8) the number of bp in the longest uninterrupted stretch of the gene; (9) the number of bp in the longest uninterrupted stretch of the gene relative to the total length of the gene; (10) the number of bp in the longest stretch of the gene interrupted by at most one insertion, relative to the total length of the gene; and (11) transposon density [14] (equivalent to the inverse of the mean number of bp between insertions). For all metrics, if there were no inserts within a gene, this gene was assigned the maximum (minimum) possible value for that metric.

We found that for both the PEC dataset and the Keio dataset, the two best predictors of essentiality were the mean distance between inserts (or density) (AUC = 0.969 for the PEC dataset, 0.95 for the Keio dataset, and 0.971 for the genes on which both datasets agreed on the essentiality classifications); and the fraction of the gene that lay in the longest uninterrupted region (AUC = 0.969 for the PEC dataset, 0.955 for the Keio dataset, and 0.971 for the genes on which both datasets agreed on the essentiality classifications) (Additional file 4: Figure S3). We selected mean distance as on average, it marginally outperformed the other statistics on the gold standard data set.

### Inference of IS element dynamics

We used 100 bp paired end Illumina sequencing data from this same library to look for structural rearrangements due to IS elements in the genome, which may result in genes appearing to have no mapped reads and thus no transposon insertions. This analysis was complicated by the fact that many IS elements share close to 100 % identity with others around the genome. For these analyses we thus restricted our searches to regions of the genome for which we had a priori expectations that they harboured a rearrangement (i.e. if there were no transposon insertions inferred, and the orthologous *E. coli* locus was non-essential or absent). Specifically, we performed the following procedure: we extracted a 50-kilobase pair (Kbp) region from the genome surrounding each hypothesized rearrangement (in all cases, this was a deletion). We then used bowtie2 with the paired end option, allowing up to 10 Kbp inserts (the default insert length is 500 bp) to map all reads from our 100 bp PE dataset. From these mapped reads, we retained only read pairs that had (1) mapping quality scores greater than 20; (2) at least one read that matched perfectly (i.e. at all 101 bases of the read) to the genome; and (3) were unique in their length at any specific location (thereby excluding artefacts such as PCR doublets). From these paired reads we then inferred the insert size, which is plotted in Additional file 12: Figure S8. The vast majority of insert sizes ranged between 100 and 400 bp. However, some were much larger (e.g. up to 9,000 bp in Additional file 12: Figure S8B). We inferred that these surrounded regions of the genome that must have been deleted.

Such deletions would result in the set of genes contained within as being inferred as essential because of their lack of transposon insertions. As such, in the vast majority of cases, we found that when large operons lacked insertions but had non-essential orthologous operons in *E. coli*, or had no orthologues in *E. coli*, these operons were in fact missing from the *Shigella* clone that we used, most likely due to the rapid dynamics of IS element mediated changes in this bacterium [61]. As an example, no sequence reads we obtained mapped to the *yeaKLMNOP* operon, which spans a total of 9,240 bp. Upon further analysis using the paired end genomic data set, we found that this region was clearly missing from our *Shigella* clone (Additional file 12: Figure S8B). This was similarly true for several other operons, as well as for single genes. We did not consider any region in which we identified a deletion in our downstream analyses.

## Additional files

Additional file 1: Figure S1. Distribution of transposon insertions across the genome. We observed little bias on the chromosomal level of insert locations. (PDF 40 kb) Additional file 2: Figure S2. Transposon insertion locations across the entire *Shigella* chromosome. Each insertion site is indicated by a vertical black line. ORFs are indicated in light green; rRNAs in light blue; and tRNAs in orange. The annotation is taken from the GenBank sequence of *Shigella flexneri* 2a 2457T. (PDF 1121 kb) Additional file 3: Table S1. Full table of gene characteristics and orthologue relationships used in the analyses. (TXT 411 kb) Additional file 4: Figure S3. ROC curves showing the predictive power of various features. To select a feature that was the best predictor of essentiality in *E. coli* orthologues*,* used only ORFs that we had data on essentiality from both the Keio and PEC studies. We then selected transposon insertion patterns that most closely match the essentiality delineations in theses studies. Specifically, we selected the feature that maximized the number of true positive “essential” genes (maximizing the sensitivity) while minimizing the number of FP (maximizing specificity). We selected from eleven non-independent features: (1) the total number of insertions; (2) the mean number of bp between insertions; (3) the median number of bp between insertions; (4) the number of bp in the 5′ end preceding the first insertion; (5) the number of bp in the 5′ end preceding the first insertion relative to the total bp in the gene; (6) the number of bp in the 5′ end preceding the second insertion; (7) the number of bp in the 5′ end preceding the second insertion relative to the total bp in the gene; (8) the number of bp in the longest uninterrupted stretch of the gene; (9) the number of bp in the longest uninterrupted stretch of the gene relative to the total length of the gene; (10) the number of bp in the longest stretch of the gene interrupted by at most one insertion, relative to the total length of the gene; and (11) transposon density [14] (equivalent to the inverse of the mean number of bp between insertions). See the Methods section for more details of this analysis. (PDF 130 kb) Additional file 5: Table S4. Table listing tRNA genes and the number of insertions in each. (TXT 1 kb) Additional file 6: Figure S4. Analogous plots to that shown in Fig. 3, for growth in minimal glucose MOPS media after (A) 24 and (B) 48 h. In both cases, we find that the shift is less pronounced than that observed for LB. (PDF 199 kb) Additional file 7: Figure S5. Elongation factor paralogues *tufA* and *tufB* appear essential in *Shigella* as compared to *E. coli*. The orthologous *E. coli* deletion strains of *tufA* and *tufB* exhibit robust growth (OD600 of 0.72 and 0.78 after 22 h in LB), but are essential by our criteria, as is the functionally related gene *lepA*. Both *tufA* and *tufB* contain insertions only at the 5′ or 3′ ends of the genes. Genes that are essential in both *E. coli* and *Shigella* are coloured in white. Those inferred as being essential in *Shigella* but for which the orthologous deletion genotypes exhibit robust growth in *E. coli* are indicated in blue. Genes inferred as essential in *Shigella* and which do not exhibit robust growth in *E. coli* are coloured in light blue. tRNA genes are indicated in dark grey. (PDF 190 kb) Additional file 8: Figure S6. The region of the genome containing the *rfb* operon is largely uninterrupted by transposon insertions. *rfbI*, *rfc*, *rfbG*, and *rfbF* are completely uninterrupted by transposon insertions; *rfbE*, *rfbA*, and *rfbC* each harbour only a single transposon insertion. None of these genes except *rfbA* have orthologous counterparts in *E. coli* K12 due to a lateral transfer event that occurred at this locus (see main text). This operon encodes genes active in O-antigen biosynthesis. Genes inferred as being essential in *Shigella* but for which the orthologous deletion genotypes exhibit robust growth in *E. coli* are indicated in blue. (PDF 177 kb) Additional file 9: Figure S7. Schematic of the primer positions used for Illumina sequencing of transposon insertions. (PDF 331 kb) Additional file 10: Table S2. List of chromosomal SNPs and indels observed in the *Shigella* strain used here that differ from the GenBank sequence NC004741. (TXT 4 kb) Additional file 11: Table S3. List of plasmid SNPs and indels observed in the *Shigella* strain used here that are different from the GenBank sequence of the *Shigella flexneri* 2a strain 301 virulence plasmid pCP301 (NC_004851). (TXT 1 kb) Additional file 12: Figure S8. Inferred fragment lengths of perfectly mapped reads across several genomic regions suggest IS-mediated deletions. For each plot, the inferred fragment lengths are arranged by increasing length (ranked on the y-axis). Thus, very long fragments are present at the top of the y-axis. Most fragments have lengths between 100 bp and 400 bp; a small number have lengths over 1000 bp or more. It is very likely that these are not the true insert sizes, but appear that way because of large scale deletions in our *Shigella* clone compared to the clone present in the NCBI genome database; see Methods for more details. (A) A region of the chromosome in which a complicated series of rearrangements has occurred, leading to paired end reads perfectly matching to different locations in this region. 45 mapped read pairs span more than 1.5 Kbp, a size that is not concordant with the majority of insert sizes. (B) A genomic region where an approximately 10Kbp deletion occurred, removing a region containing the *yeaKLMNOP* operon. 92 mapped read pairs span more than 8.5Kbp. This region is flanked by two IS elements. (C) A region where an approximately 4Kbp deletion occurred, removing two genes with no *E. coli* K12 orthologues. 68 mapped read pairs span more than 4 Kbp, and again this region is flanked by two IS elements. (D) A genomic region where an approximately 2Kbp deletion occurred, removing *yhdW*. 244 mapped read pairs span more than 2Kbp, and the region is flanked by two IS elements. (E) A deletion in the region of the chromosome containing *S4145 (yiaN)*. 232 mapped read pairs spanned more than 1.8 Kbp, and this region is also flanked by two IS elements. (F) A region of the chromosome containing the *rfb* operon. Most of the genes within this operon are uninterrupted by transposons. However, we find no evidence that this is due to a deletion of this region in our *Shigella* clone, as we find no reads mapping across the region; a small number of reads map within the region; and the closest IS elements are 15 Kb upstream of *rfbJ* and 20 Kb downstream of *rfbA*. The genes in this operon have no orthologues in *E. coli* K12. (PDF 219 kb)

## Abbreviations

Bp: Base pairs
*E. coli* – *Escherichia coli*: BW25113
ORF: Open reading frame
PEC: Profiling the *E. coli* Chromosome database
*Shigella*: *Shigella flexneri* 2a 2457T
