# Supplementary figures and images for: Combining Shigella Tn-seq data with gold-standard E. coli gene deletion data suggests rare transitions between essential and non-essential gene functionality
Source: BMC Microbiol. 2016 Sep 6;16(1):203. doi: 10.1186/s12866-016-0818-0 (PMC5011829; doi:10.1186/s12866-016-0818-0)

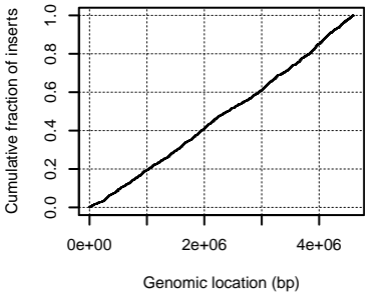

Supplement: Additional file 1: Figure S1. — Distribution of transposon insertions across the genome. We observed little bias on the chromosomal level of insert locations. (PDF 40 kb) [file 12866_2016_818_MOESM1_ESM.pdf]

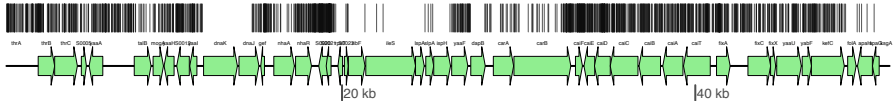

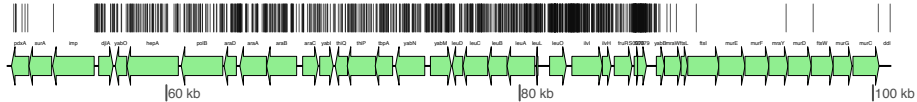



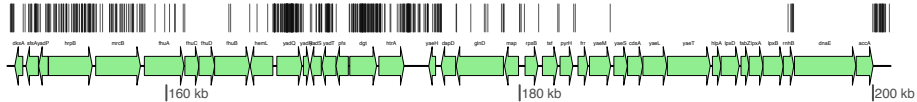



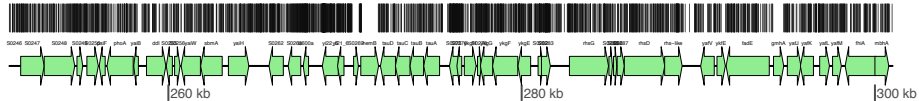





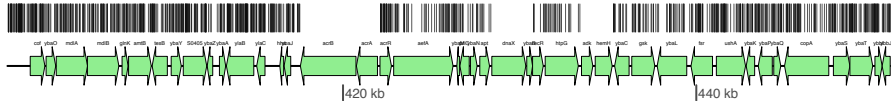

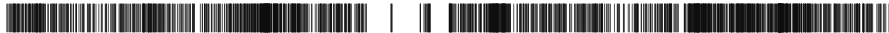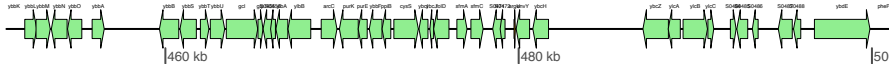

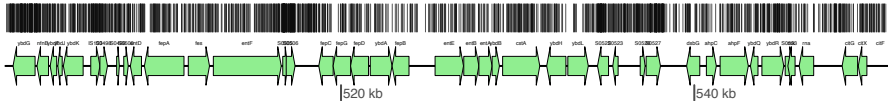

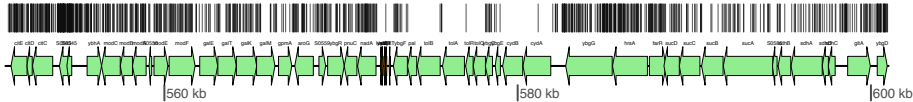

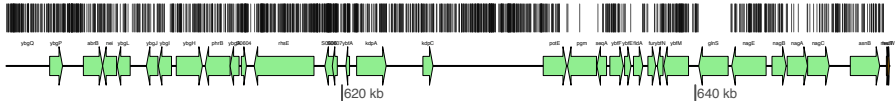

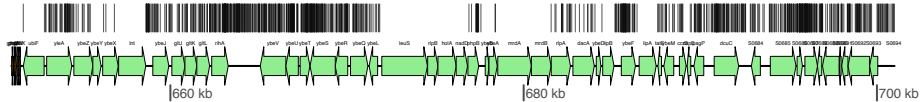

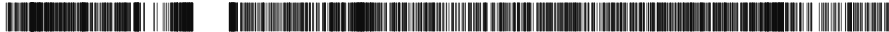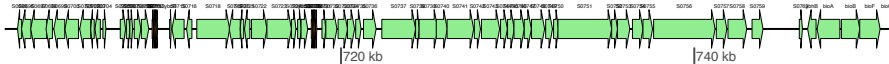

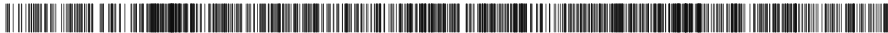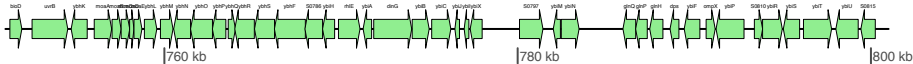

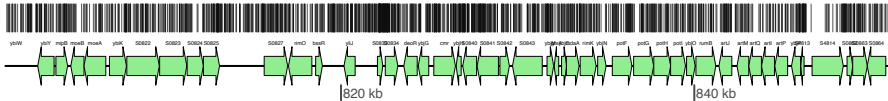



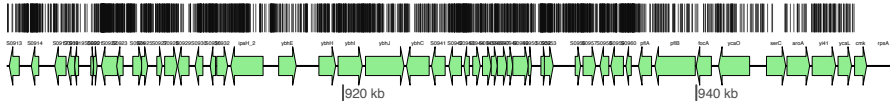

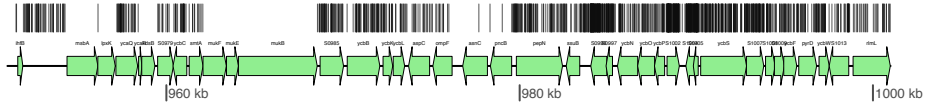

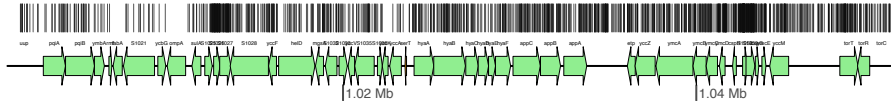



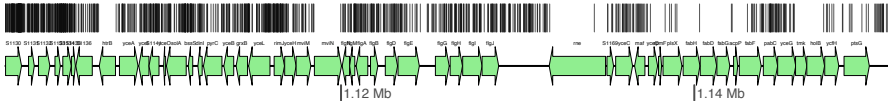

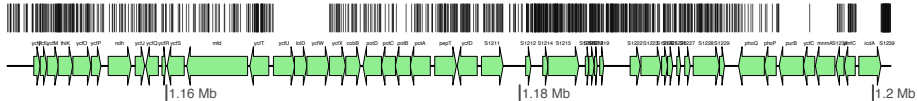

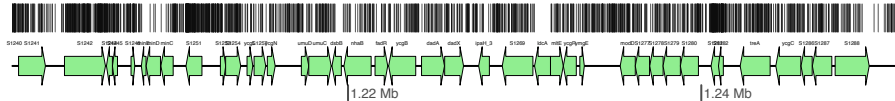

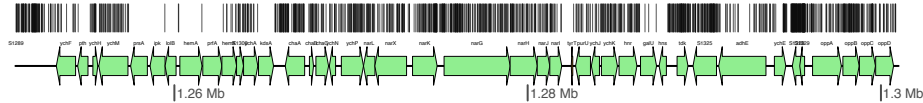

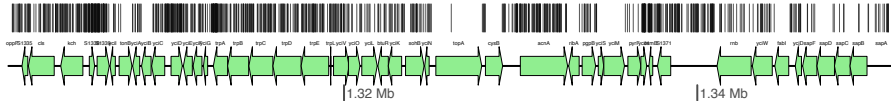

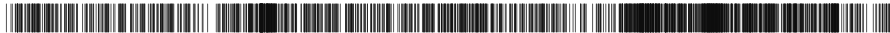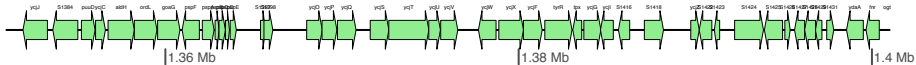

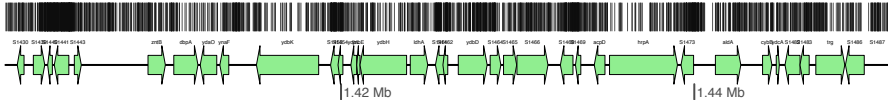



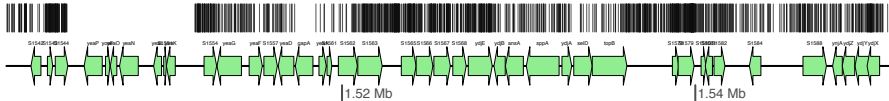

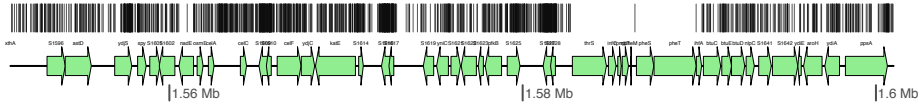

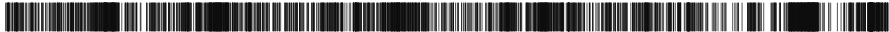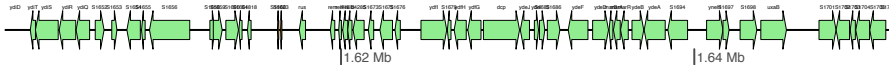

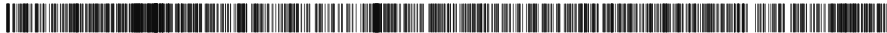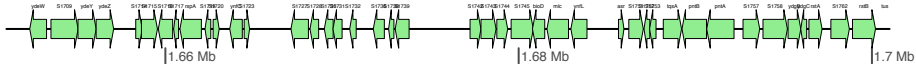

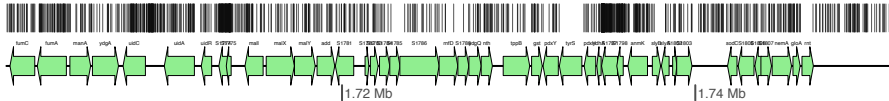

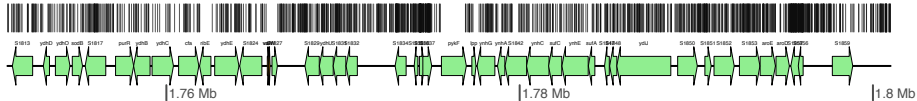

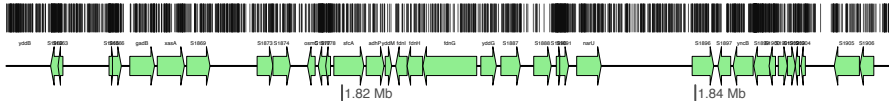

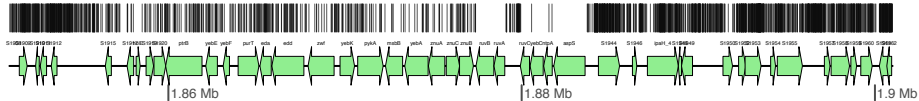

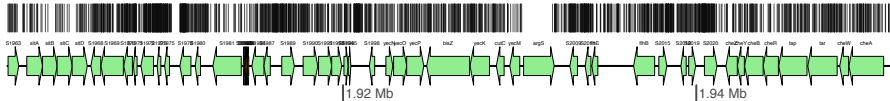

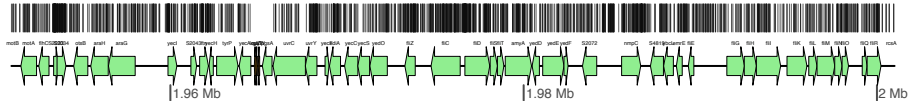



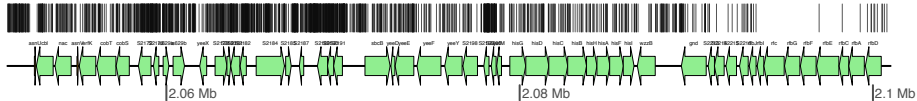

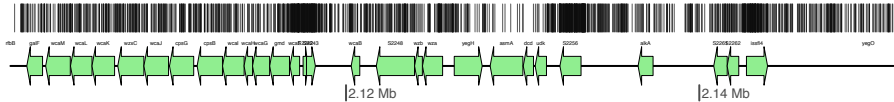

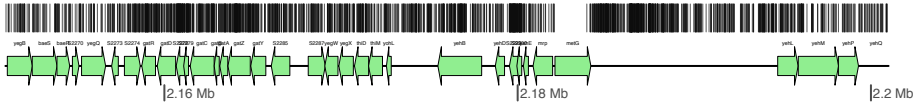

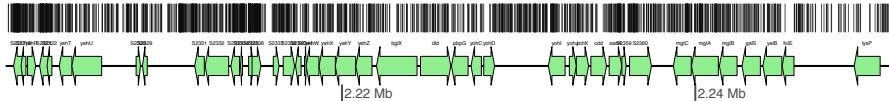

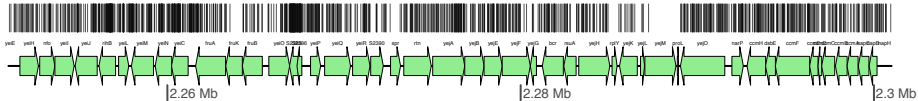

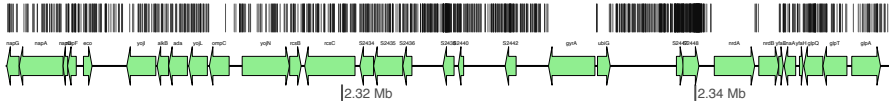

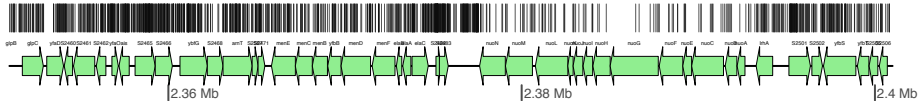

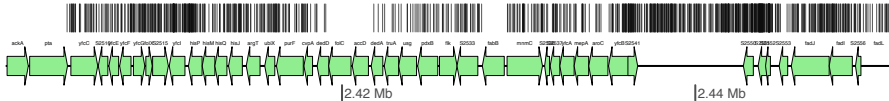

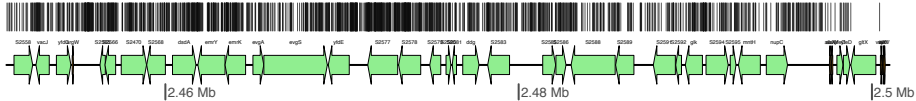



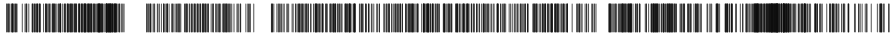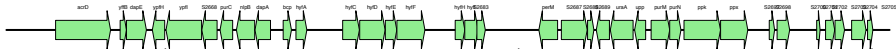

2.56 Mb

2.58 Mb

2.6 Mb

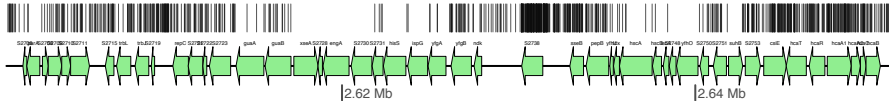

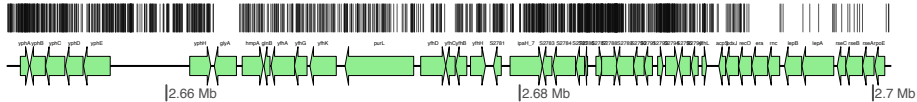



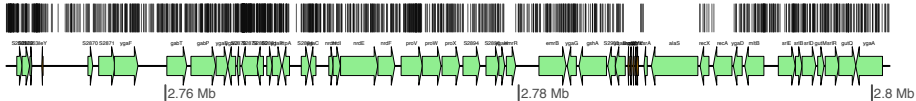

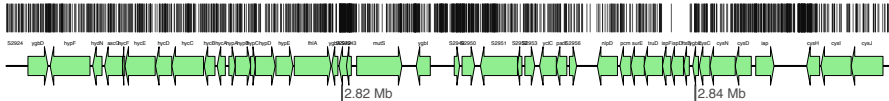

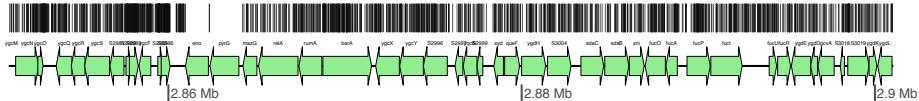

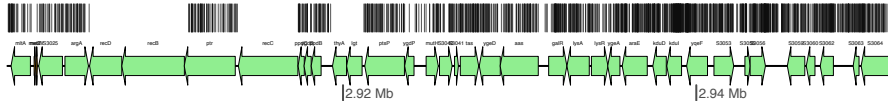

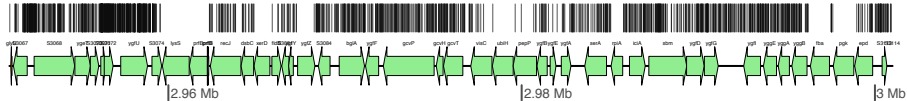

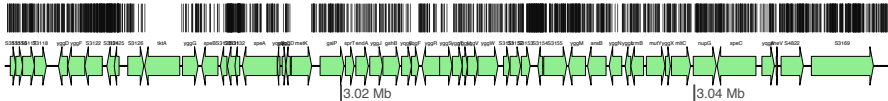

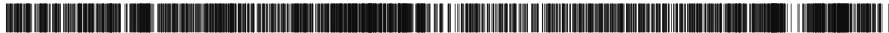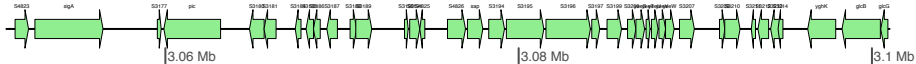

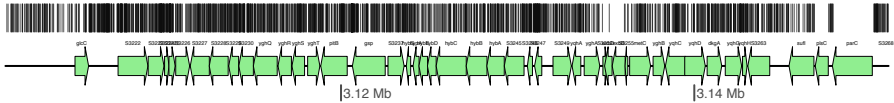

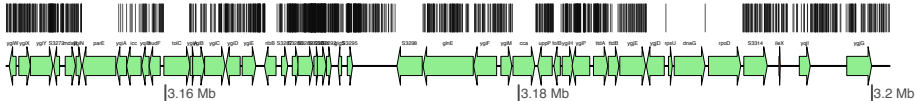

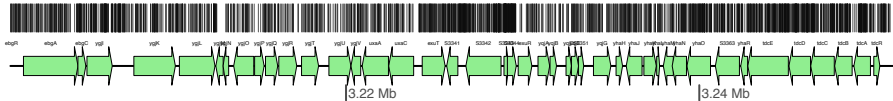





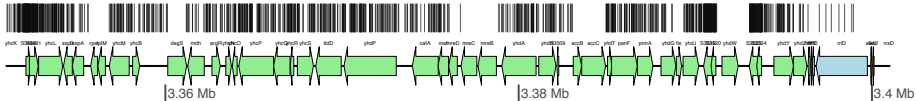

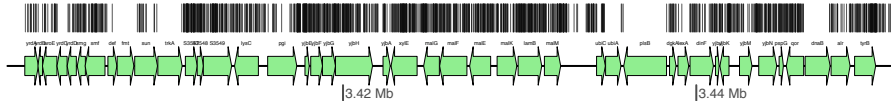

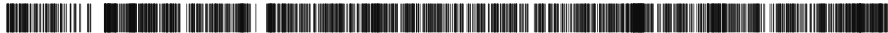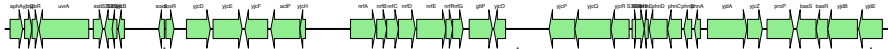

3.46 Mb

3.48 Mb

3.5 Mb

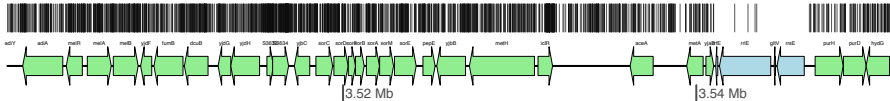



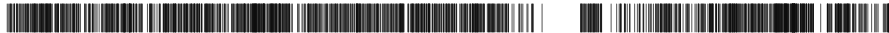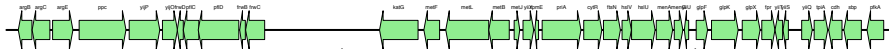

3.62 Mb

3.64 Mb

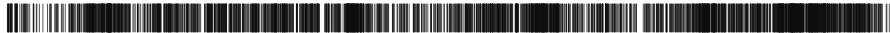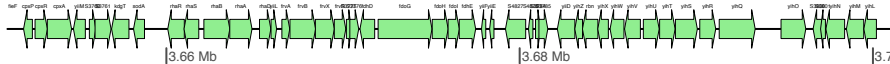

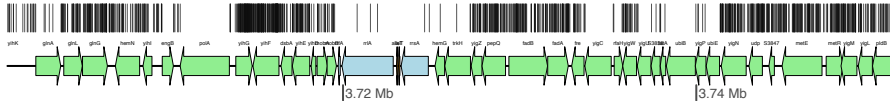



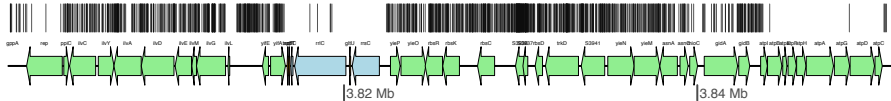

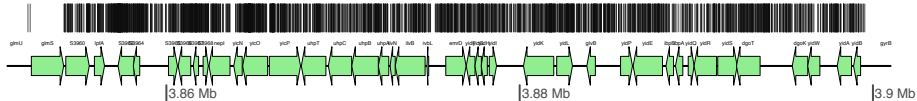

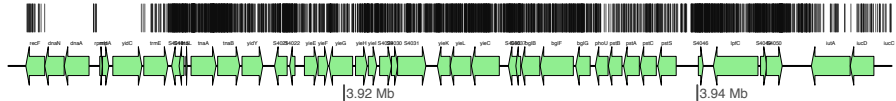

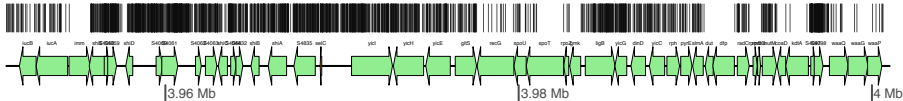

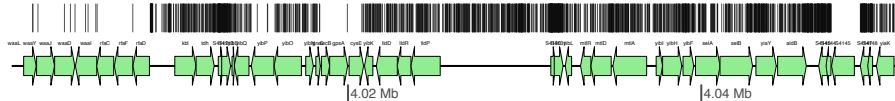

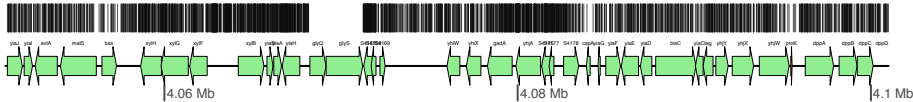

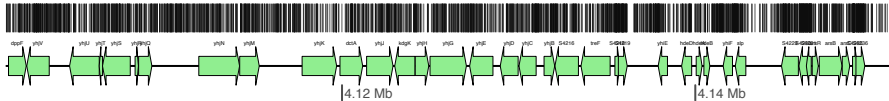

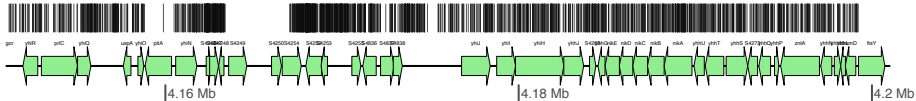

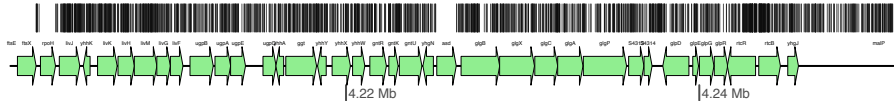

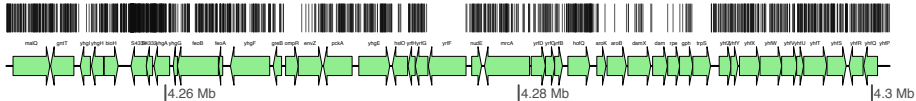



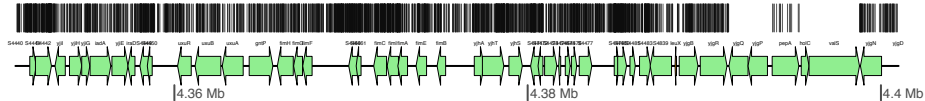

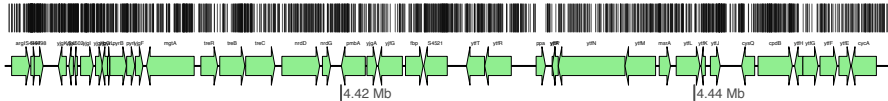



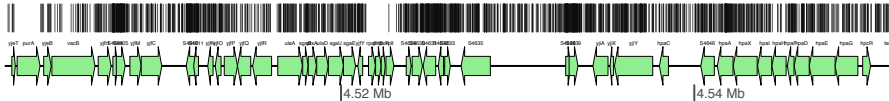

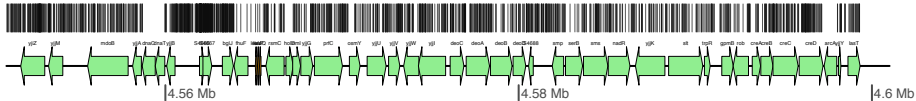

Supplement: Additional file 2: Figure S2. — Transposon insertion locations across the entire Shigella chromosome. Each insertion site is indicated by a vertical black line. ORFs are indicated in light green; rRNAs in light blue; and tRNAs in orange. The annotation is taken from the GenBank sequence of Shigella flexneri 2a 2457T. (PDF 1121 kb) [file 12866_2016_818_MOESM2_ESM.pdf]

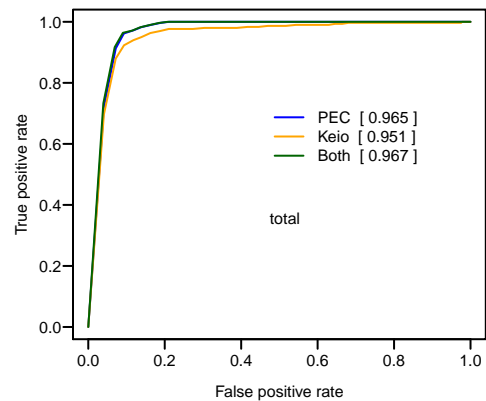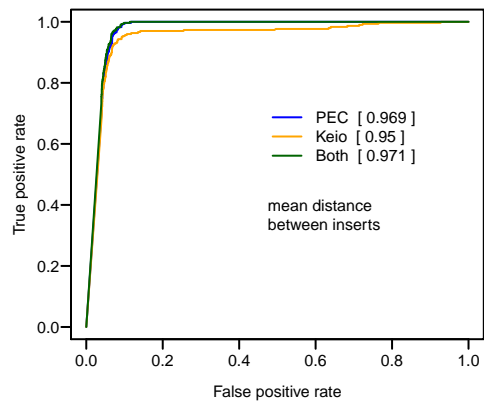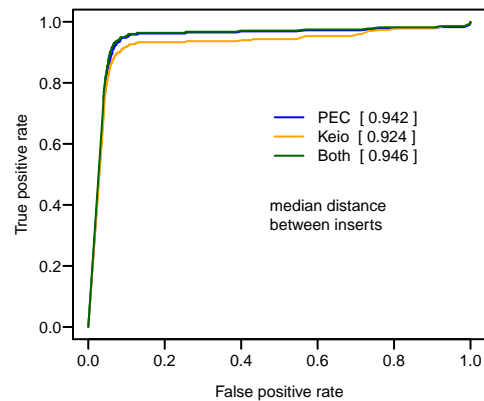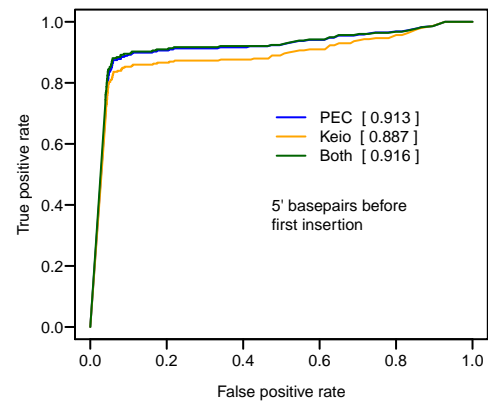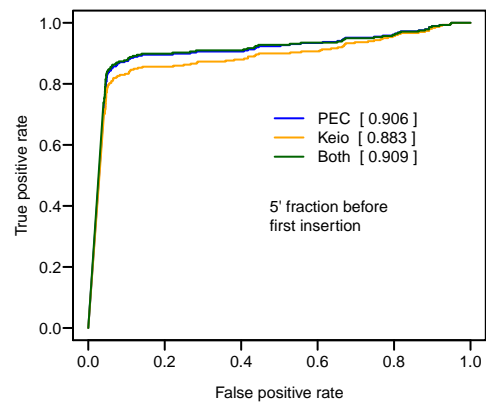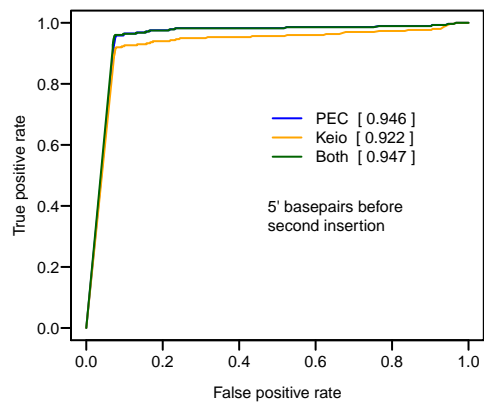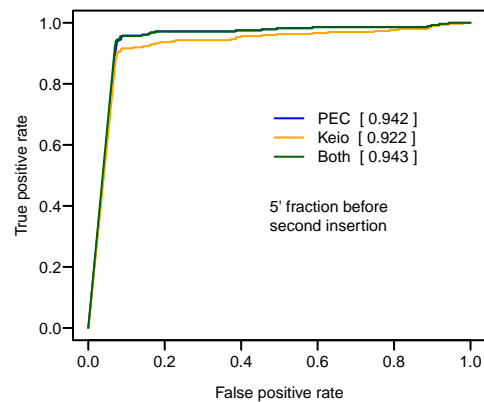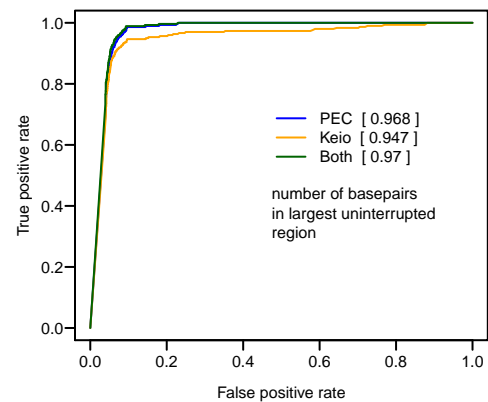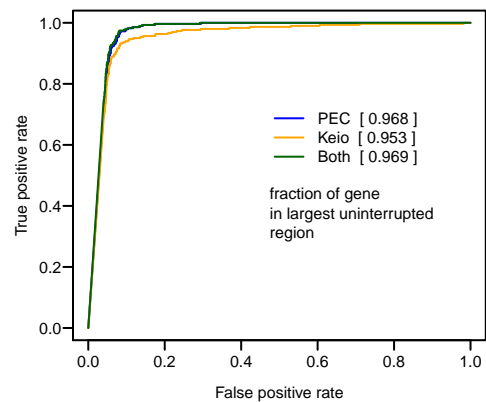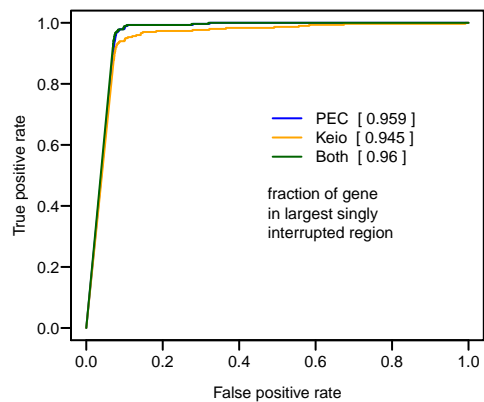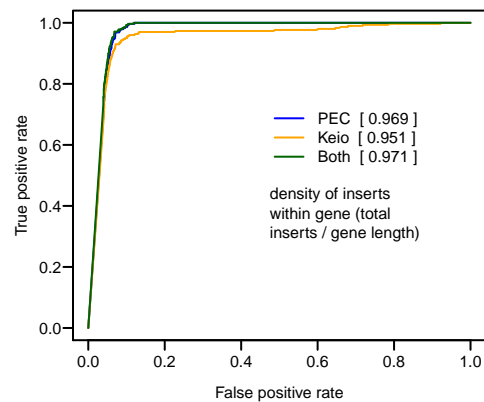

Supplement: Additional file 4: Figure S3. — ROC curves showing the predictive power of various features. To select a feature that was the best predictor of essentiality in E. coli orthologues, used only ORFs that we had data on essentiality from both the Keio and PEC studies. We then selected transposon insertion patterns that most closely match the essentiality delineations in theses studies. Specifically, we selected the feature that maximized the number of true positive “essential” genes (maximizing the sensitivity) while minimizing the number of FP (maximizing specificity). We selected from eleven non-independent features: (1) the total number of insertions; (2) the mean number of bp between insertions; (3) the median number of bp between insertions; (4) the number of bp in the 5′ end preceding the first insertion; (5) the number of bp in the 5′ end preceding the first insertion relative to the total bp in the gene; (6) the number of bp in the 5′ end preceding the second insertion; (7) the number of bp in the 5′ end preceding the second insertion relative to the total bp in the gene; (8) the number of bp in the longest uninterrupted stretch of the gene; (9) the number of bp in the longest uninterrupted stretch of the gene relative to the total length of the gene; (10) the number of bp in the longest stretch of the gene interrupted by at most one insertion, relative to the total length of the gene; and (11) transposon density [14] (equivalent to the inverse of the mean number of bp between insertions). See the Methods section for more details of this analysis. (PDF 130 kb) [file 12866_2016_818_MOESM4_ESM.pdf]

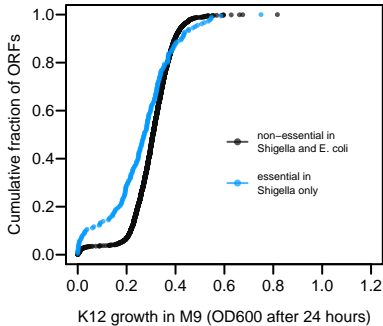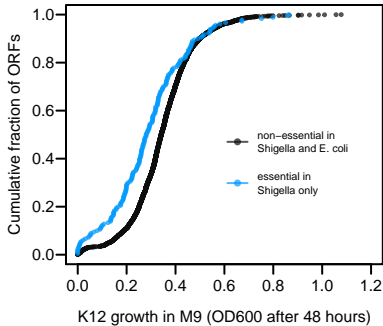

Supplement: Additional file 6: Figure S4. — Analogous plots to that shown in Fig. 3, for growth in minimal glucose MOPS media after (A) 24 and (B) 48 h. In both cases, we find that the shift is less pronounced than that observed for LB. (PDF 199 kb) [file 12866_2016_818_MOESM6_ESM.pdf]

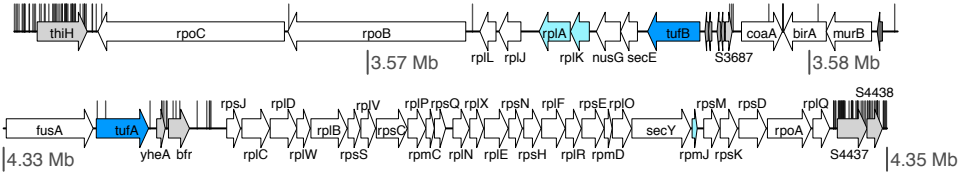

Supplement: Additional file 7: Figure S5. — Elongation factor paralogues tufA and tufB appear essential in Shigella as compared to E. coli. The orthologous E. coli deletion strains of tufA and tufB exhibit robust growth (OD600 of 0.72 and 0.78 after 22 h in LB), but are essential by our criteria, as is the functionally related gene lepA. Both tufA and tufB contain insertions only at the 5′ or 3′ ends of the genes. Genes that are essential in both E. coli and Shigella are coloured in white. Those inferred as being essential in Shigella but for which the orthologous deletion genotypes exhibit robust growth in E. coli are indicated in blue. Genes inferred as essential in Shigella and which do not exhibit robust growth in E. coli are coloured in light blue. tRNA genes are indicated in dark grey. (PDF 190 kb) [file 12866_2016_818_MOESM7_ESM.pdf]

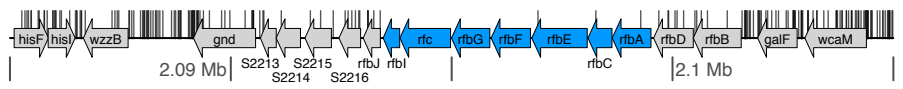

Supplement: Additional file 8: Figure S6. — The region of the genome containing the rfb operon is largely uninterrupted by transposon insertions. rfbI, rfc, rfbG, and rfbF are completely uninterrupted by transposon insertions; rfbE, rfbA, and rfbC each harbour only a single transposon insertion. None of these genes except rfbA have orthologous counterparts in E. coli K12 due to a lateral transfer event that occurred at this locus (see main text). This operon encodes genes active in O-antigen biosynthesis. Genes inferred as being essential in Shigella but for which the orthologous deletion genotypes exhibit robust growth in E. coli are indicated in blue. (PDF 177 kb) [file 12866_2016_818_MOESM8_ESM.pdf]

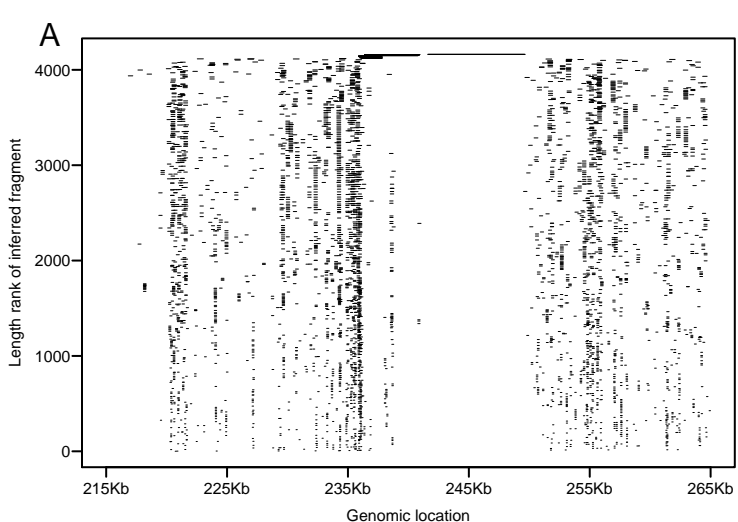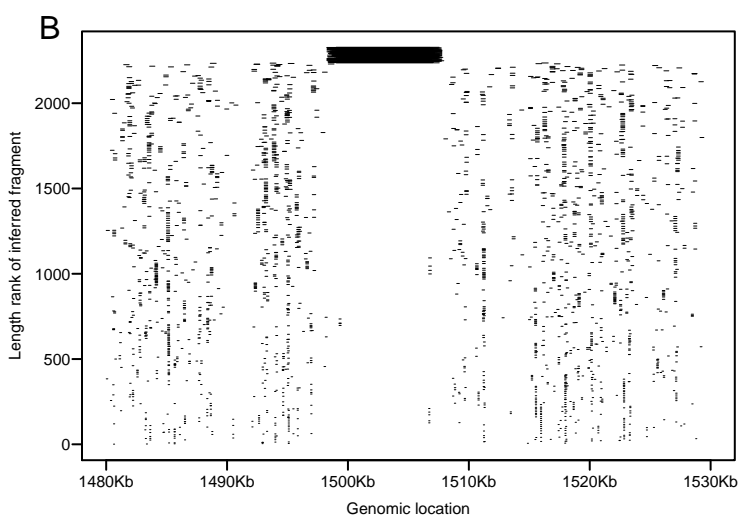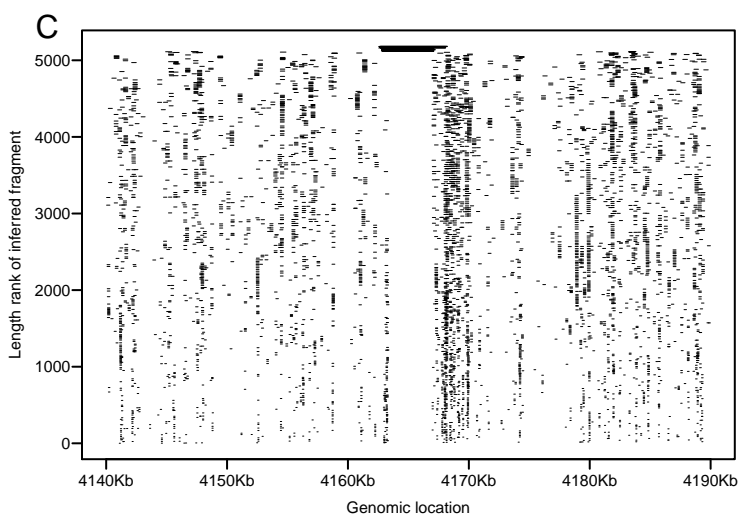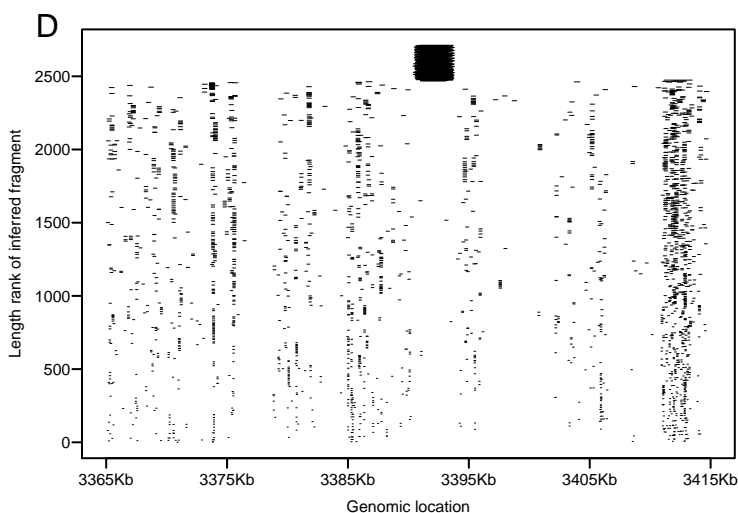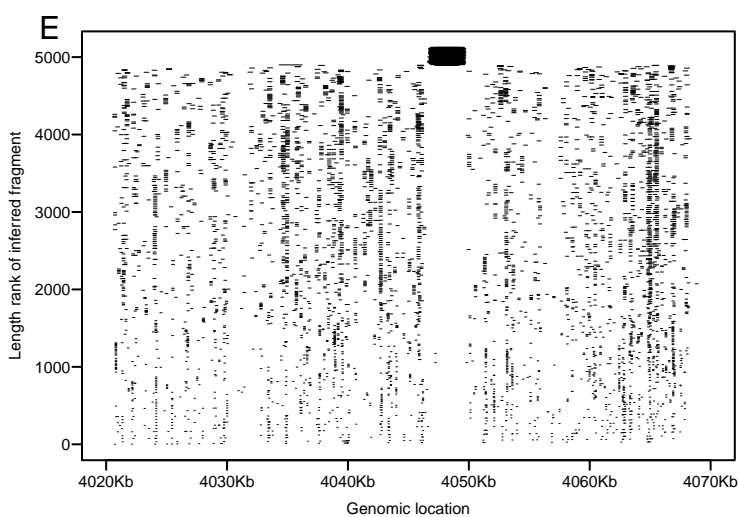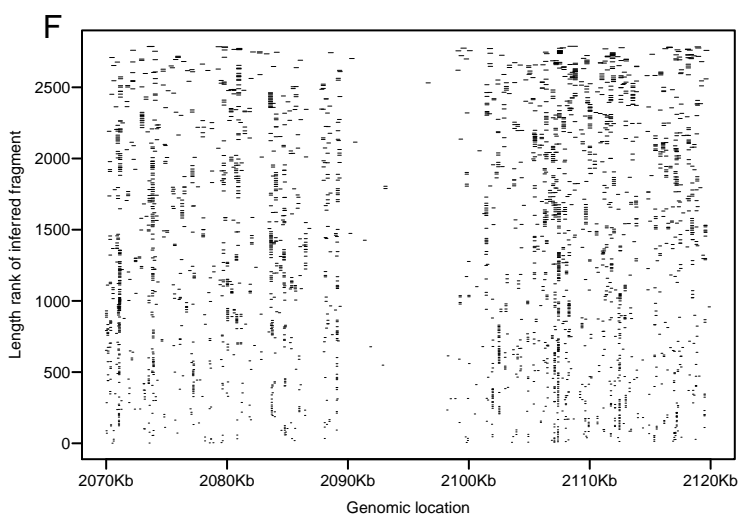

Supplement: Additional file 12: Figure S8. — Inferred fragment lengths of perfectly mapped reads across several genomic regions suggest IS-mediated deletions. For each plot, the inferred fragment lengths are arranged by increasing length (ranked on the y-axis). Thus, very long fragments are present at the top of the y-axis. Most fragments have lengths between 100 bp and 400 bp; a small number have lengths over 1000 bp or more. It is very likely that these are not the true insert sizes, but appear that way because of large scale deletions in our Shigella clone compared to the clone present in the NCBI genome database; see Methods for more details. (A) A region of the chromosome in which a complicated series of rearrangements has occurred, leading to paired end reads perfectly matching to different locations in this region. 45 mapped read pairs span more than 1.5 Kbp, a size that is not concordant with the majority of insert sizes. (B) A genomic region where an approximately 10Kbp deletion occurred, removing a region containing the yeaKLMNOP operon. 92 mapped read pairs span more than 8.5Kbp. This region is flanked by two IS elements. (C) A region where an approximately 4Kbp deletion occurred, removing two genes with no E. coli K12 orthologues. 68 mapped read pairs span more than 4 Kbp, and again this region is flanked by two IS elements. (D) A genomic region where an approximately 2Kbp deletion occurred, removing yhdW. 244 mapped read pairs span more than 2Kbp, and the region is flanked by two IS elements. (E) A deletion in the region of the chromosome containing S4145 (yiaN). 232 mapped read pairs spanned more than 1.8 Kbp, and this region is also flanked by two IS elements. (F) A region of the chromosome containing the rfb operon. Most of the genes within this operon are uninterrupted by transposons. However, we find no evidence that this is due to a deletion of this region in our Shigella clone, as we find no reads mapping across the region; a small number of reads map within the region; and the closest IS e [file 12866_2016_818_MOESM12_ESM.pdf]
