# Supplementary material for: Combining Shigella Tn-seq data with gold-standard E. coli gene deletion data suggests rare transitions between essential and non-essential gene functionality
Source: BMC Microbiol. 2016 Sep 6;16(1):203. doi: 10.1186/s12866-016-0818-0 (PMC5011829; doi:10.1186/s12866-016-0818-0)
Supplement: Additional file 9: Figure S7. — Schematic of the primer positions used for Illumina sequencing of transposon insertions. (PDF 331 kb) [file 12866_2016_818_MOESM9_ESM.pdf]

1: Arbitrary PCR

using phusion PCR enzyme, so that no A's added at 3' end

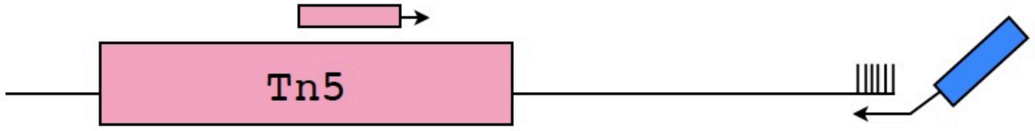


```
1_transp_deep_seq_F1: TCTATCGCCTTCTTGACGAG
```

```
1_transp_deep_seq_R2: GTGACTGGAGTTCAGACGTGT GCTCTTCCGATCT NNNNNNNNNNggtgc
1_transp_deep_seq_R3: GTGACTGGAGTTCAGACGTGT GCTCTTCCGATCT NNNNNNNNNNgatat
1_transp_deep_seq_R4: GTGACTGGAGTTCAGACGTGT GCTCTTCCGATCT NNNNNNNNNNagtac
```

2: Nested PCR (addition of P7 and P5)

using phusion PCR enzyme, so that no A's added at 3' end

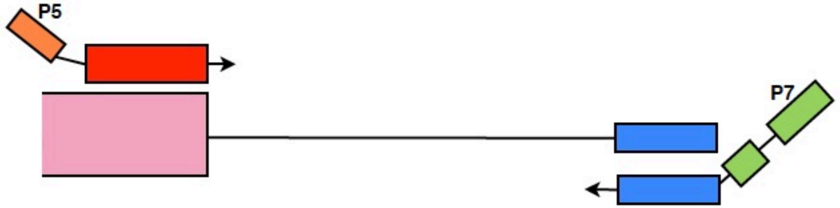


```
2_transp_deep_seq_ FP5: AATGATACGGCGACCACCGAGATCT actggtcggcg CATTAGGGGATTCATCAG
```

```
Rev_P7 6_02
CAAGCAGAAGACGGCATACGAGAT agtctt GTGACTGGAGTTCAGACGTGT
CAAGCAGAAGACGGCATACGAGAT gtatct GTGACTGGAGTTCAGACGTGT
CAAGCAGAAGACGGCATACGAGAT tcatgg GTGACTGGAGTTCAGACGTGT
CAAGCAGAAGACGGCATACGAGAT cgcgac GTGACTGGAGTTCAGACGTGT
CAAGCAGAAGACGGCATACGAGAT acgata GTGACTGGAGTTCAGACGTGT
CAAGCAGAAGACGGCATACGAGAT tataga GTGACTGGAGTTCAGACGTGT
```

3: Sequencing on Illlumina chip

```
transp_deep_seq_Custom seq 1: actggtcggcgCATTAGGGGATTCATCAG
```

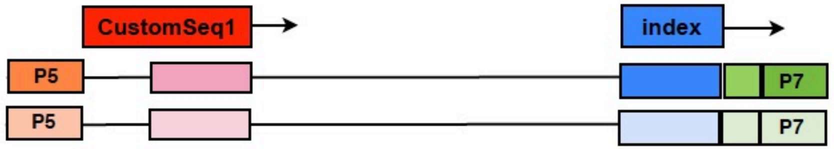

double stranded PCR product,
gel purify band/smear at (300-400bp)
(insert at 200-300bp)

4: Barcode is read on Illlumina chip

```
transp_deep_seq_Index seq: GATCGGAAGAGCACACGTCTGAACTCCAGTCAC
```
